# Looking at people looking at art: observations of art interactions in an everyday urban environment

**DOI:** 10.3389/fpsyg.2025.1658946

**Published:** 2025-09-08

**Authors:** Anna Lena Knoll, Jan Mikuni, Eva Specker

**Affiliations:** ^1^Department of Cognition, Emotion, and Methods in Psychology, Faculty of Psychology, University of Vienna, Vienna, Austria; ^2^Leibniz-Institut für Wissensmedien, Tübingen, Germany; ^3^Department of Psychology, Science Faculty, University of Tübingen, Tübingen, Germany

**Keywords:** aesthetics, art, passers-by, urban environment, observational study, art interactions, social engagement, everyday life

## Abstract

**Introduction:**

Placing art in urban spaces can make urban public environments more attractive and colorful by offering beautiful and restorative environments. This may invite people to spend time in the area and create opportunities for social engagement, and community development. In this observational study we collaborated with “Keine Galerie” (translating to “not a gallery”), a small window gallery in the city of Vienna (Austria) to address the following questions: Does the presence of publicly available art influence people's behavior (in terms of type, frequency, and duration) in an urban space? Does it enhance peoples' social interactions, such as the amount of conversations in a group?

**Methods:**

To capture the impact of presence of art, we collected data during two exhibitions by two different artists at Keine Galerie (i.e., art conditions) as well as between exhibitions when no art was visible (i.e., control condition). We used observational methods to unobtrusively assess how pedestrians who were passing through the study area interact with their environment either with or without art.

**Results:**

Our results showed that art being present invites passers-by to interact with the space more than when no art is present (no art vs. art conditions) but also that the type of art may matter (Ex.1 vs. Ex. 2), which influenced not just the amount of interactions but also which interactions took place.

**Discussion:**

We discuss these quantitative and qualitative differences, also with regard to potential confounding factors (e.g., weather), and propose avenues for further research into the impact of art in public space.

## 1 Introduction

A city's look can influence, for instance, how we move through the city—for example, more interesting looking facades slow us down and promote human interaction (Gehl et al., 2006).

This aesthetic aspect of urban environments—specifically cultural elements such as art—has been shown to be a powerful design tool to promote space perceptions, making them more exciting and beautiful (Motoyama and Hanyu, 2014; Pogrmić and Ðercan, 2021), and even promote wellbeing (e.g., Trupp et al., 2025). Public art interventions have been shown to reduce the feeling of anxiety, stress, and negative mood (Mikuni et al., 2024). This not only includes visual artworks but also extends to performance art such as musical busking, which not only influences how we aesthetically perceive the area the performance takes place in but can also lead to feeling (sonically) restored (Ho and Szubielska, 2024; Ho and Au, 2021).

Moreover, the presence of street art can influence perceptions of neighborhood safety, friendliness, noisiness, and cleanliness, particularly in neighborhoods with lower socioeconomic standing (Estrada Gonzalez et al., 2025). Relatedly, publicly accessible art may strengthen people's connections to the city, and their neighborhoods (Kühnapfel et al., 2025). This increase in connectedness could counteract feelings of loneliness, one of various urban lifestyle factors contributing negatively to the wellbeing of the urban population (Ventriglio et al., 2021). Despite these recent findings, modern cities have become gray and boring (Thinks Insight, 2022), with buildings becoming larger (Gehl et al., 2006) and more uniform. If we continue to make all modern cities look similar, they will increasingly lose their identity, their uniqueness (Gehl et al., 2006; Pogrmić and Ðercan, 2021), and may lose their potential to raise the quality of city life. Thus, given increasing urbanization (United Nations, 2018), it is a pressing social task to design cities with people in mind, as city design impacts the behavior, social connection, and wellbeing of their residents.

Maybe luckily, cities are not static entities, especially not in modern times:

“Cities are changing faster than ever before, so much that many buildings and infrastructures become under-used or empty since they are no longer able to serve their users' needs.” (Interreg Europe, 2024)

Turning this rapid change into the right direction may help us create better cities for people to not just reside, but to flourish in. One way to do so is by leveraging the power of aesthetic interventions. By changing how a city looks, it may be possible to change how the city feels. Cities have many spaces that offer themselves to art; There is a long history of various forms of street art and graffiti, as well as planned art in public spaces (most commonly statues or monuments). However, as the above quote suggests, many spaces in urban environments may remain unused; We can transform such (temporarily) unused spaces to give art a space in different ways—buildings that are entirely or partly empty may host studios, exhibitions, and events (for a local Viennese example, see never-at-home.at); empty store fronts and office windows can display art in a way that lets everyone who passes by experience it.

“Keine Galerie” (“not a gallery,” translated from German), a small unconventional gallery space in Vienna, builds on this philosophy. Rather than being a traditional gallery space that requires people to come inside to see the art, it puts the gallery “outside” on the street. This is implemented by placing the artworks in the unused store fronts in what is now an office space. Thus, transforming empty store windows into an outside gallery space making art accessible to everyone (see instagram.com/keine.galerie and reachguys.com/projects/keine-galerie). Art displayed in spaces that are visible from street level may make art accessible to those who do not usually go to museums or galleries. It takes away the boundary of having to “step in” to the gallery, and instead makes the art “step out” to the street. Though such initiatives are not common place yet, there is already initial research supporting the potential of such interventions—at least in terms of fostering community and connection to one's neighborhood (Kühnapfel et al., 2025).

In this paper, we collaborated with Keine Galerie to build on these foundations and to see to what extent such art interventions can change the behavior of casual passers-by on the street. Our aim was to investigate how people interact with such freely accessible art in a naturalistic manner. Thus, when art “steps into” our everyday lives, where we may be busy with our day-to-day tasks, does it impact us? Can it actually change our behavior? For instance, does it make us slow down, leading us to spend more time in this part of the city? And does it matter which type of art is exhibited?

To assess these questions, we directly compared the same urban environment either with art present or without art present, to have a naturalistic control setting. The incorporation of the control condition is important as it allows us to more specifically attribute eventual effects to the presence of artworks. This is especially relevant as previous work on art interactions, as Trupp et al. (2025) point out in their scoping review, often lacks a control condition (see also Kühnapfel et al., 2025; Mikuni et al., 2024).

In addition, we wanted to investigate the role specific artworks themselves play—is any art sufficient or do different kinds or styles of art lead to different behavior? Therefore we compared two different art exhibitions (held in the same space, i.e., Keine Galerie). This allows us to address aspects often limiting generalisability and stimuli-specific effects, specifically variation in stimuli which can be particularly challenging in field experiments.

Keeping these aspects in mind, we compared whether behaviors observed in our study area differ when different sets of artworks are present, as well as when no artwork is present. We thus performed three sets of observation during two different art exhibitions featuring artwork of one artist each and a no-art control condition.

Furthermore, in our everyday life we often are not on our own. We may meet up with friends at a café, pick up our children from school, etcetera. The people around us and our interactions with them may influence how attentive we are to and in turn how we interact with and perceive our environments in general (Shteynberg, 2015), and in urban (Staats and Hartig, 2004), or art environments (Christidou, 2016; Reitstätter and Christidou, 2024; Christidou, 2018), specifically. For instance, a child may excitedly point at a dog that the parents would not have otherwise paid any attention to. Thus, we compared whether people interact with their environment differently when they are alone or in a group and whether this depends on art being present or not.

In contrast to previous work (e.g., Kühnapfel et al., 2025; Mikuni et al., 2024) we take an observational approach. This approach was explicitly chosen as our goal was to study people's behavior as they were naturally passing through our study area, being unaware of being measured and without limitations on the duration and the way of interaction with the testing location. Though this approach comes with its own limitations (human errors in the tracking of behavior, difficulty in quantifying/assessing inter-rater reliability, no way to constrain confounding factors, etc.), it can effectively address limitations of other approaches. To be specific, other approaches either recruit people and place them specifically in these environments or recruit passers-by to actively participate in the study. Both of these approaches may lead to interactions with the art being different than when people would encounter the same art naturally in their own time, walking through a city. For example, in our day-to-day lives, we may be more constrained, e.g., if we need to pick up a child from school, we may have limited time to stop and look at the art. These constraints are not present when people are specifically there to participate in the study (and thus have a specific time reserved for this participation) which may lead to both longer and different interactions. Furthermore, for people recruited on the scene, these constraints may bias the sample that is recruited, i.e., people who have time constraints will not agree to participate, whereas the ones that have time will agree to participate. Again, leading to a potential bias in the behavior that is observed. Further biases may occur due to participant's awareness of being observed or restrictions placed on their behavior (e.g., being forced to stay within a certain area). In other words, the study settings described above possess high ecological validity, but art interactions occurring in these settings may still contain bias. Along these lines, Ho and Szubielska (2025) suggest that in everyday settings people generally act in “life-mode” as opposed to “art-mode.” In the general life-mode people will go about their daily life, not paying attention to art that may be present around them. Ho and Szubielska (2025) suggest that a switch to “art-mode” is necessary for people to engage with art that is present in their everyday life. While such a switch may occur for various reasons, participants specifically recruited for a study will typically receive some information on the purpose of the study and will therefore more readily switch to art-mode than somehow just going about their daily life. Thus, while these approaches have their merits and can meaningfully contribute to our knowledge, they also have their limitations—as does the observational method. With our approach, we wanted to counter some of these limitations of past approaches by prioritizing the assessment of naturalistic behavior (accepting the limitations inherent in this approach).

To disrupt the natural flow of people's everyday lives as little as possible we opted to focus specifically on observable behaviors. A list of behaviors to track was devised looking at museum visitor studies, as well as in agreement with Keine Galerie. While we can look at museum studies for behaviors that people are likely to engage in when encountering art in an everyday urban environment, these behaviors may not necessarily translate one to one from the museum to a street environment. This will not only affect what interactions we observe but also how we observe them. For instance visit duration can in a museum be tracked as the time between leaving and entering a specific room; this already widely differs between people (e.g. Serrell, 1997). People may quickly go from one room to the next or spend a longer time in a specific room. In contrast, in our study space, visits are not constrained by the walls of a room, people may wander off to different parts of the square Keine Galerie is located in, then decide to come back to the art or leave the area entirely. This makes it difficult to decide when a “visit” to the study area is finished, thus potentially making global visit duration a somewhat less accurate measure than it is in museum studies. We track when people enter and leave the general study area (see Section 2.2), as well as additional behaviors taking place within that time. The most obvious behavior is probably looking at the art works, this can range from a quick glance to stopping in front of the art and taking it in for a long time. Even in museums, viewing times tend to be rather short (Smith and Smith, 2001; Smith et al., 2017). Furthermore, in an everyday environment people may be more constrained by people needing to go somewhere else. Thus, those who need to be elsewhere (can) only give a quick glance to the art; those who are not in a rush to go elsewhere can take this time to view the art, stop and look at the it in similar ways as they would in a museum. We here track both the number of looks that are given to the artworks, as well as how long people look at an artwork while stopping in front of it. Stopping, although not necessarily required, can lead to further engagement such as reading labels, taking photos etcetera (Rodriguez-Boerwinkle and Silvia, 2025). Further, since our study took place in an everyday setting where people do not necessarily come to seek out art specifically, we tracked behaviors that are not related to the art present in the space. Some of these will also be found in a museum setting (e.g., people using their phones, e.g., Reitstätter et al., 2022), while others are not likely to occur in a museum (e.g., stopping/sitting down to smoke). We tracked these to get an idea of how much of time in the area people spend on art vs. on other things. The full list and further explanations on these behaviors within our study setting specifically can be found in Section 2.4.

To sum up, the present study explores how people interact with art in their everyday urban environment by tracking behaviors passers-by engage in when passing through our study area when art is present vs. when not. While there is a lack of previous research in this direction, we did have a few expectations regarding people's behavior. First, we expected that people passing through the area would show differences in the time spent in the area as well in the frequency of tracked behaviors, for instance they may stop more often and look toward the gallery space more, when art is present. Further, we expected some differences in observed behaviors between groups and people alone, given that one person in a group could for instance point out art being present. Our aim was to provide first insight into how art in urban settings may impact behavior which can then be built on using confirmatory approaches in the future, as well as show how observational methods can be a valuable tool for the study of urban aesthetics.

## 2 Methods

### 2.1 Ethics statement

This study was purely observational and we did not collect any personal identifying information about any of our observed subjects, thus following the Helsinki declaration, no ethical approval was necessary for this study.

### 2.2 Study area

**Figure 1:**
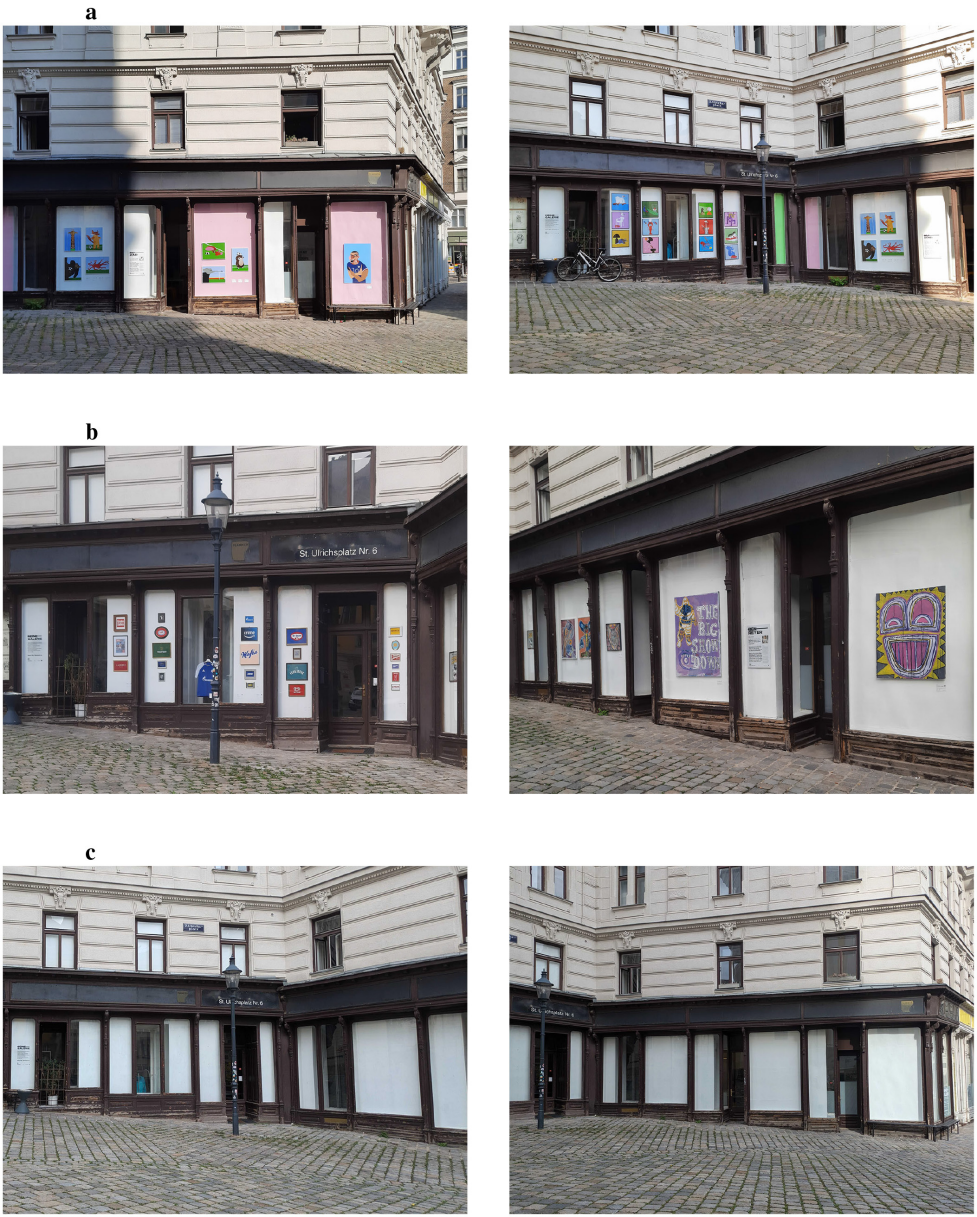
Exhibition conditions: **(a)** Exhibition 1, **(b)** Exhibition 2, **(c)** Control.

**Figure 2:**
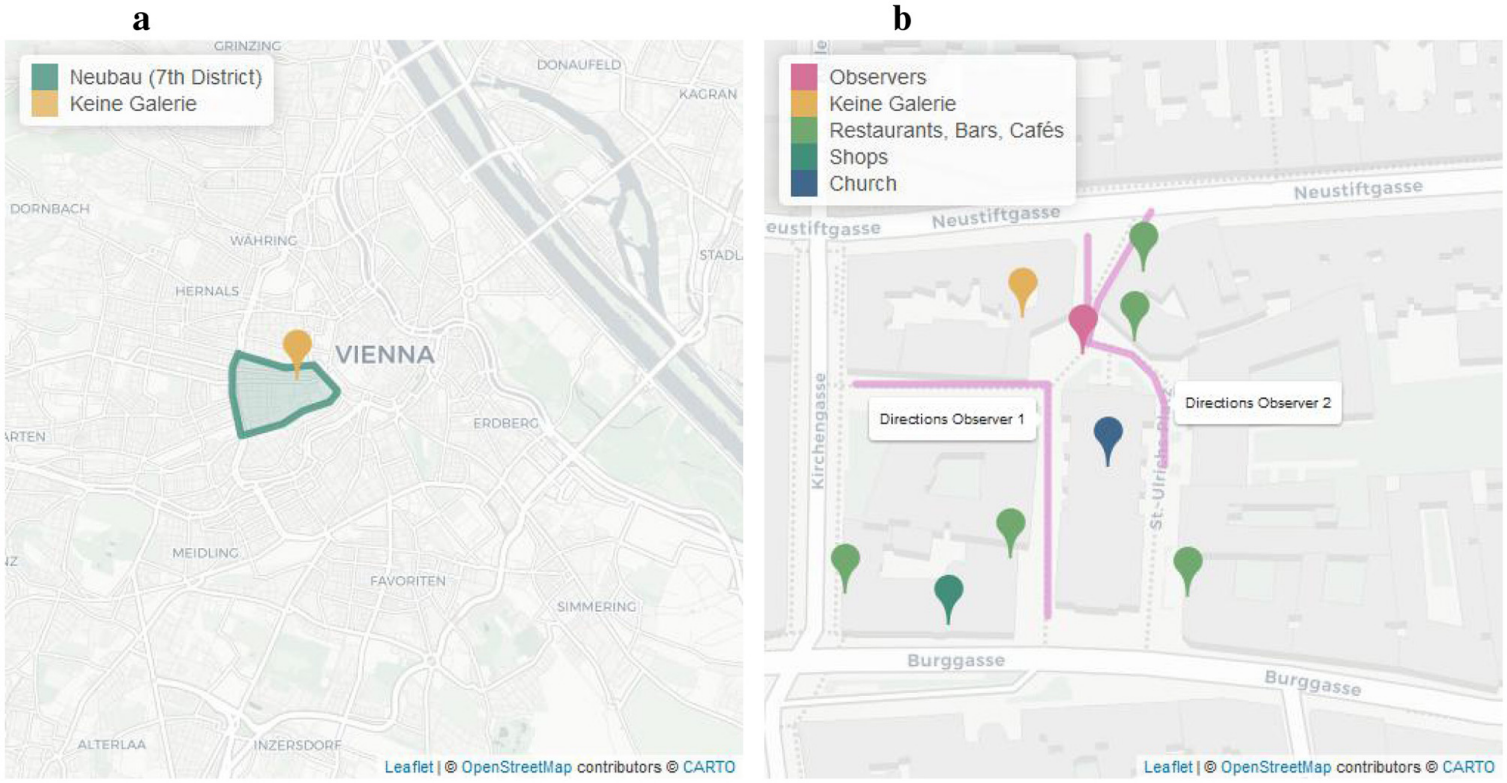
**(a)** Location of Keine Galerie within Vienna. **(b)** The study area at St. Ulrichsplatz with location of Keine Galerie, observers, and some additional points to illustrate the area.

The observations took place outside Keine Galerie (hereafter, KG). KG uses the shop windows (see Figure 1b) that are part of their office building as a small gallery space where local artists present their work. Their first exhibition took place in September 2023, roughly one year before our data collection. While some people may know about KG, it is, currently, by no means “famous.” Hence, most people are unlikely to come for a targeted visit to KG specifically, making it an ideal space to observe how people interact with art they naturally encounter in an everyday urban space. KG is located at St. Ulrichsplatz (see Figure 2b, showing KG and the surrounding St. Ulrichsplatz) in Neubau, the centrally located 7th District of Vienna (see Figure 2a, showing the location of KG within Vienna). This district has a population of around 31.500 people, with a relatively low rate of unemployment (in 2024: 59 per 1,000 people) and a medium average income (in 2022: 29.378€), compared to the whole of Vienna (unemployment: 103/1,000 people, average income: 26.005€) (Land Wien, 2024a,b). It is considered to be a lively, creative part of Vienna (Land Wien, 2024a), with many cafés, vintage clothing shops etcetera. The study area is also within a 5–10 minute walk from multiple museums, thus our sample of passers-by likely included not only residents but also a relatively high number of tourists.

### 2.3 Data collection periods

We conducted observations during two art exhibitions and a no art control condition. After our initial observation session on 4th September 2024 (1st observation day, Exhibition 1, observation times: 9.50am–12.50pm and 1.50pm–4.30pm), we decided to conduct our observations from 9.50am–12.30pm and 3pm–5.30pm. These hours seemed to cover a reasonable amount of busier (e.g., around 4.30–5pm) and less busy times (e.g., 10–11am), allowing us to get a good mix of people who might be passing the area for various reasons (e.g., at busier time points adults who were picking up their children from school; people taking their dogs for walks during less busy times). Due to rain, we did not stick to these observation hours on 10th October (1st day at Exhibition 2), here we instead ended our observations at 11.15am.

#### 2.3.1 Exhibition 1

Exhibition 1 (“Am Platz,” see Figure 1a) featured artwork by M. Ali Ziaei from the series “Animals & Design” (*n* = 12, 50 × 50cm), Animals & Football (*n* = 7, 50 × 70cm), as well as two caricatures of football players Marko Arnautovic and Kylian Mbappé (80 × 120cm, for more details see instagram.com/p/C-FLSM-sKhs/?hl=de). The start of this exhibition was timed with the 2024 European Football Championship in June; our observations took place at the end of the exhibition in early September (4–6th September 2024).

#### 2.3.2 Control

Between the two exhibitions the gallery was left empty (see Figure 1c), this served as our control condition. Observations took place on 25–26th September 2024. Note that large labels about the gallery remained and that the neighboring office also features art and a mirror that passers-by may pay attention to (see Supplementary Figure S1).

#### 2.3.3 Exhibition 2

Exhibition 2 (“Who We Are,” Figure 1b) featured artwork by Ben Reyer who creates socially critical collages with elements of pop culture, advertising, and print media. Artworks varied in sizes, from the 3 larger ones on the right-hand windows, to 6 medium sized works (50 × 60 cm or 50 × 70 cm), to 23 smaller works of varying sizes and shapes (see instagram.com/p/DBtEBWjNcfQ/?hl=de). Due to weather conditions, observations were split between the first (10–11th October 2024) and second half (25th October 2024) of October.

#### 2.3.4 Note on weather conditions during observations

The weather differed between the three sets of observations (see Supplementary Figure S2 for temperatures during observations). With Ex. 1 taking place in early September, we still had summer temperatures, however between Ex. 1 and the Control the weather changed quite drastically. This delayed when we were able to make our observations; The Control was originally planned straight after Ex. 1, but due to severe rain with flooding throughout large parts of Austria, needed to be pushed back to the end of September. By the time we were able to make our observations for Ex. 2, temperatures had further decreased, and it remained rainy. The weather not only affected when we were able to make our observations, but potentially also the behaviors people engaged in. We will refer back to this potential influence in the results and discussion.

### 2.4 Measures

#### 2.4.1 Tracked behaviors

Behaviors to track were decided on in agreement with KG, considering both our and their interests to devise a list of behaviors that were (1) theoretically relevant, (2) likely to occur and, (3) feasible to track. The final list (see Table 1) includes both behaviors that are performed to interact with the art, as well as behaviors that are non-art related. This allows some comparison between people who are simply passing through the area (e.g., just walking) and people who additionally engage in some other types of behavior.

**Table 1:** Behaviors, modifiers, and information about subjects that we tracked.

**Behaviors**	**Modifiers**	**Type**	**Changes**	**Definitions**
Walk by	-	State	-	Walking past KG from any direction
Bike past	-	State	Added after Ex.1	People ON bikes; includes scooters, delivery people
Stop	(Not) looking at art, eating	State	-	-
Going inside	-	State	-	Going inside KG office
Photo	Of art, with art, of something else, QR/AR scan	Event	-	-
Talk	To each other, on the phone, to observers, to KG employees	Event	“To observers” added after Ex. 1	-
Reading	Labels, phone, something else	Event	“Phone” modifier added after Ex1	
Looking	-	Event	-	Looking at art specifically
Pointing	-	Event	-	Pointing at art specifically
Gender	Age (child, teen, 20s, 30s, 40s, 50s, 60s, 70+)	Event	-	Tracked as behavior (gender) + modifier (age) (assumed from looks); Large groups (e.g., school classes): often noted group size only as they often passed through the area too quickly to track age/gender of everyone
Works there	-	Event	Added after Ex1	Tracked as behavior; People working in the area pass by KG often

First off, to be able to contextualize frequency of behaviors as well as the potential impact of such interventions, we were interested to establish a baseline of people who pass by the exhibition space. This not only determines, to a large extent, the total sample size of subjects but can also be used to establish meaningful percentages to interpret the more specific behaviors that occur. Therefore, we tracked both people who walked by as well as cycled past. We did not track people passing through in cars, because KG is located on a square with limited car access. Observations of how people pass through the space are also used to calculate the duration of their visit, i.e., time of when they first arrive to the time they leave the area. This is somewhat less accurate than tracking visit duration in a museum as we do not have specific rooms as a boundary that can act as the start and end of a visit.

Besides how people pass through the space, we tracked more specific behaviors that may indicate interest in either the art present in the study area or interactions with other elements within the study area. These were primarily behaviors that are common in museum visitor studies, as well as relevant in a non-museum setting such as ours (e.g., stopping: Serrell, 1997; reading labels, looking, phone use, taking photos: Reitstätter et al., 2022; stopping, social interactions, phone use: Yalowitz and Bronnenkant, 2009; photos, selfies specifically: Piancatelli et al., 2021, pointing: Christidou, 2018, 2013).

To measure engagement with the art, we assessed if people looked at the art. Note that especially in our setting, looking can occur without stopping, e.g., someone on their way to work, may not have the time to stop to look at the art in detail but may still look at the art in passing. Theoretically it would be interesting to look at, for instance, smiling behavior to assess potential affective impact of these brief encounters. But, smiling can be very subtle or even imperceivable for human observers which makes it hard to reliably track within an observational paradigm therefore smiling (and similarly subtle behaviors), were excluded for feasibility reasons. We revisit the potential impact of these brief encounters in the discussion.

To contextualize viewing behavior further, we tracked stopping. Here we differentiated whether people stopped to look at art or for other reasons. Due to the natural setting, some people used the square to, for instance, sit down and eat their lunch or they stopped to looked at e.g., the church. Thus, to properly measure engagement with the art a differentiation was necessary between engaging in a behavior for the purpose of art interaction or for non-art related reasons.

In addition, to go beyond viewing behavior, we assessed if people took pictures or were reading—here we were specifically interested in whether they took photos of the art or read the labels accompanying the artworks/exhibition. Nonetheless, given the real-life setting, we noticed during Ex. 1 that many people also read (or looked at) their phone, and thus also tracked this behavior specifically in Ex. 2 and the control condition. Similarly, with respect to taking pictures, we differentiated between if people took pictures of the environment (e.g., of the church or square) or if they took a picture of the artworks/exhibition. As only the latter indicates engagement with the art/exhibition. Notably, KG also provides a QR code that can be scanned to unlock an AR experience, thus we also specifically tracked if people scanned this, but this was very rare (see Supplementary Figure S5).

Finally, to assess the social dimension, we tracked if people talked to each other, went inside the KG office to talk to the exhibition organizers, and if they engaged in pointing behavior. For pointing behavior, like looking behavior, we only tracked if people pointed at the art/exhibition and not at other parts of the environment. For talking behavior, we tracked who people talked to (i.e., to other people if they were a group, on the phone, to the KG employee, to the observers). Adjustments to the list (as the example of reading on the phone above) were made after the first set of observations (Ex. 1) to add things we did not consider when coming up with the original list (see Table 1 for changes made).

#### 2.4.2 Other tracked variables

Besides behavior we also tracked if people were passing alone or in a group, as well as assumed age and gender. As a precise measure of these variables would be impossible with our method, we divided age in broad categories (i.e., child, teen, 20s, etc.) and used only a binary category for gender (male, female). Both were tracked based on how people looked, and should thus be treated with caution. In the case of very large groups (e.g., school class) often only group size was noted due to practical limitations such as the group passing through the area too quickly to note everyone's age and gender. Based on Ex. 1 we also added a qualifier for people who worked in the area (people from neighboring offices, cafés, etc.) as we could clearly see them e.g., taking a smoking break. We did not exclude them from the sample. Although their behavior may be different from casual passers-by, they may equally be impacted by the art exhibition.

### 2.5 Observational set-up and procedure

Observations were recorded using Behayve (Android v4.4.3, behayve.com/) installed on three Android phones (personal phones of ALK and JM, and a lab-owned phone). Behayve logs timestamps for different behaviors that are pre-defined by the user when creating a study in the app. Additionally, behaviors can be specified through “Modifiers,” for instance, one of our recorded behaviors was taking photos for which we set the modifier options “of art,” “with art,” “of something else,” and “QR or AR scan” (see above). Some behaviors are set as “event” behaviors, some as “state” behaviors. For event behaviors, one timestamp is recorded for each instance of the corresponding behavior; for state behaviors, Behayve records two timestamps (i.e., start and end time). State behaviors need to either be stopped once the person is no longer engaged in this behavior or has left the study area, or they are stopped automatically by the start of another behavior that is mutually exclusive (e.g., walking and stopping). We additionally recorded information about the people we observed (i.e., whether the subjects work in the area and assumed gender and age) as behaviors. During observation, the observers need to manually select each behavior that occurs by tapping the corresponding button within the app.

The study was set up in Behayve as follows: (1) A new study was created using the app on ALK's phone; (2) We pre-defined all behaviors we wanted to track. This included setting the behavior type (i.e., state or event) and any “Modifiers” to the behaviors we wanted to track (e.g., “of art” as modifier for taking a photo); (3) The study data base was exported from ALK's phone and imported on JM's and the lab owned phone. Screenshots of our set-up and the study set-up database, which can be used to recreate the study set-up on any phone with Behayve installed, can be found on OSF (osf.io/35e9y/).

#### 2.5.1 Procedure

On each observation day, two observers were present. Observers sat on chairs in front of the church opposite the gallery space (see Figure 2b). This location provided the best view of both the gallery and people passing through the area from different directions. At the beginning of each observation day, observers agreed on the direction they were responsible for (see Figure 2b). Adjustments to the agreed upon directions were made if there were multiple subjects coming from the same direction; here one observer communicated to the other observer whether they needed help tracking one of multiple subjects. Note that during busy times, with many people coming from each direction, we were not always able to track everyone due to only 2 observers being present at any given time but a varying amount of people passing through the area at different times. For instance if there are many people passing through in a relatively short time span (e.g., a person who simply passes by the KG and does not engage in any way will pass within a matter of seconds) a few may have been missed by the observers.

**Figure 3:**
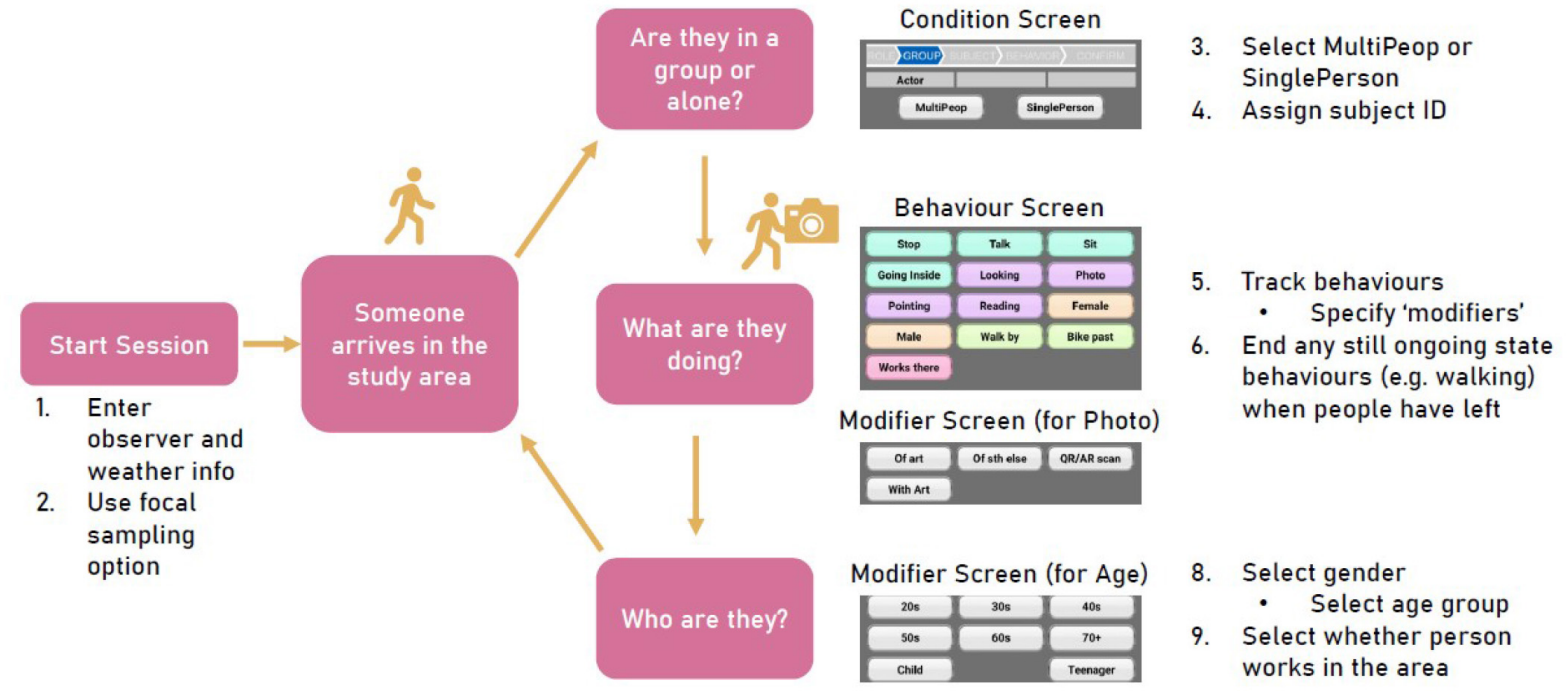
Illustration of observation procedure.

To start observations, observers opened the Behayve app on their phones (see Figure 3 for illustration), selected the relevant study and from there started an observation session. Here, the first step is inputting observer name and weather information (temperature, cloud cover, short description of weather). After a session and the focal sampling mode was started, the observers waited for people to arrive in the area. As soon as someone entered the space, clearly heading the direction of the gallery, their behaviors were tracked by one of the observers. This is done by tapping the buttons in the Behayve app that correspond to the observed behaviors

First, we selected whether they were in a group or alone and assigned a subject ID (note that all people in a single group were tracked with the same subject ID). The first behavior tracked was typically how the person/group moved through the space (i.e., walking or cycling). Next, the responsible observer waited for other behaviors to occur and tracked each instance of them occurring. The observers stopped all ongoing state behaviors as soon as the corresponding subject left the study area. Gender, age, and whether the subjects work in the area were recorded either right after the corresponding subject left the study area or while they were not engaged in any behaviors of interest.

### 2.6 Analysis

We analyzed data in R (R v4.2.0, R Studio v2024.12.1.563, R Core Team, 2022; RStudio Team, 2022) employing the GGplot2 package (Wickham, 2016) for data visualization. Maps were created with the leafleat (Cheng et al., 2024) and sf packages (Pebesma, 2018) using data for Vienna district boundaries found here github.com/codeforgermany/click_that_hood/blob/main/public/data/vienna.geojson.

To investigate how the presence of art and being alone or in a group influences our interactions with the study area, we ran Quasi-Poisson regressions on the number of tracked interactions. Poisson regressions were chosen as our dependent variables represent count data which typically follows a poisson distribution more closely than a normal distribution (Elhai et al., 2008). Further, our data was overdispersed, meaning that the assumption of variance being equal to the mean of the count data was not met. To take this into account Quasi-Poisson Regressions were used.

The first three (Model 1–3) had identical categorical predictors: Exhibition condition (i.e., Ex. 1 vs. Ex. 2 vs. Control; baseline = Control) and Social condition (i.e., single vs. group, baseline = group). The dependent variable differed between the three models: Model 1) total number of any type of art interaction (i.e., looking, pointing, stopping to look at art, sitting down to look at art, taking photos of art or scanning QR/AR codes, reading labels, and talking to someone from KG), Model 2) total number of looking only, and Model 3) total number of all art interactions that were not looking. Combining the different types of art interactions this way allows to us to take into account levels of art engagement to some extent, with the interactions included in Model 3 potentially indicating more extended level of engagement than “only” looking at the art. Next, we extended these three models by including mean-centered temperature as an additional predictor (Models 4–6).

As these models get tend to get more complicated to interpret with additional predictors, we wanted to reduce this complexity by looking at the influence of weather, separately for each Exhibition condition. Thus we split our data by Exhibition condition and ran models with the predictors Social condition and temperature (mean-centered per exhibition condition) for each. We again did this for all 3 dependent variables above, resulting in 9 additional models (see Supplementary Tables 2–4).

Data and analysis scripts are available on OSF (osf.io/wh5uz/).

## 3 Results

### 3.1 Observed passers-by demographic information

We observed a sample of 4,813 subjects. Note that some people may have passed by more than once (e.g., in the morning and later in the afternoon), however as remembering each individual passing was practically impossible, they were treated as separate subjects. Thus some people may be counted multiple times.

Looking at each exhibition condition, the sample was slightly bigger during Exhibition 1 (1,802, Ex.2: 1,544, Control: 1,467) likely because the total observation hours were slightly more and the weather (see Supplementary Figure S2) was better than during Exhibition 2 and the Control condition.

**Figure 4:**
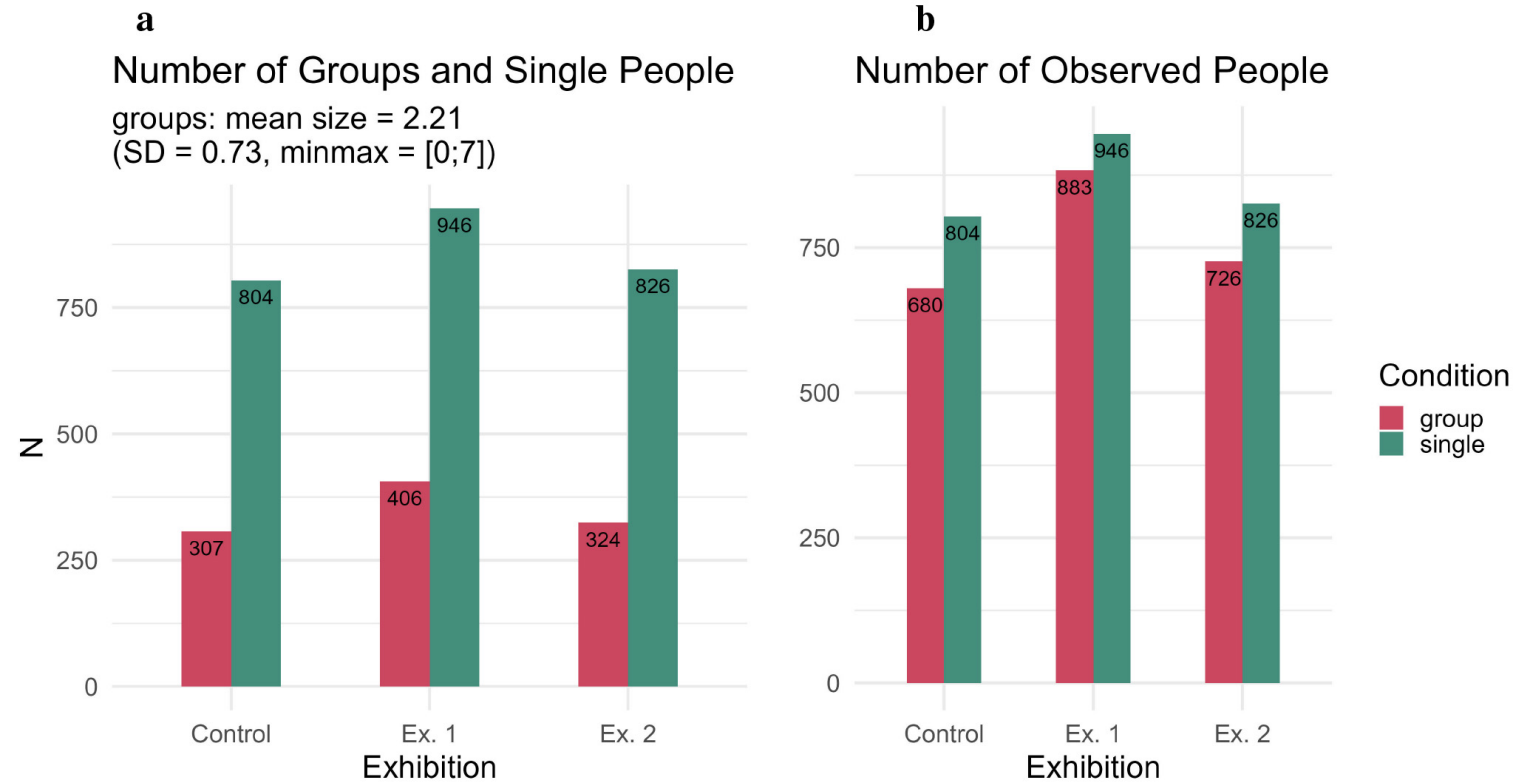
Number of Observed People. We observed fewer groups than people alone **(a)**, however taking group size into account shows that the total number of people in groups is only slightly less than the number of people who were alone **(b)**.

Overall, the total number of groups was lower than the number of people who were alone (see Figure 4a). To further take group size into account we summed up how many people were in each group. As we did not track group size specifically and each member of a group was tracked as the same subject, this was done by using the recorded information on gender as gender was tracked once per person in the group (missing gender information for 27 groups). By doing so, we see that while there were fewer groups than people alone overall, the total number of people in these groups is similar to the total number of people who were alone (see Figure 4b).

**Figure 5:**
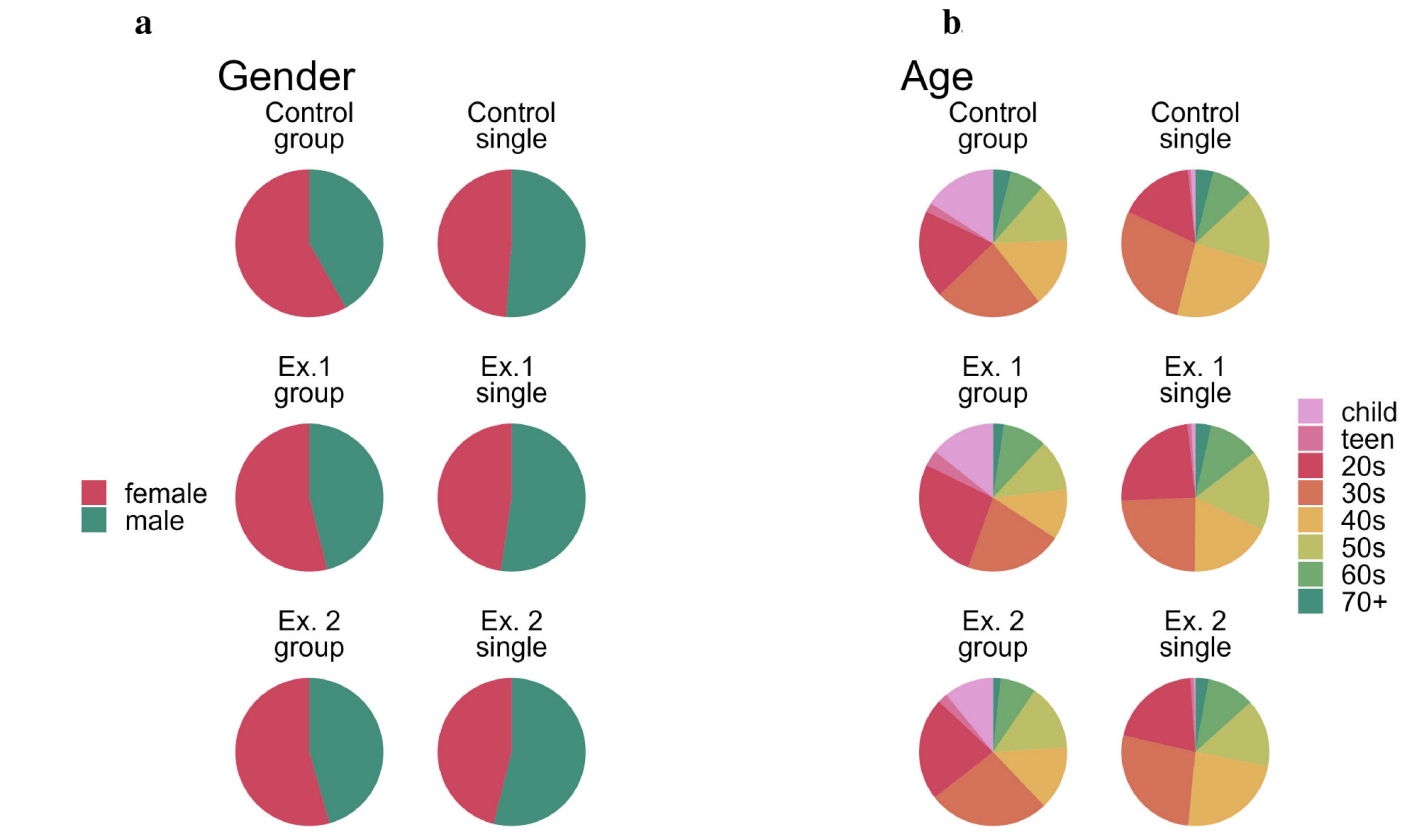
**(a)** Gender and **(b)** age of observed subjects.

Gender distribution was roughly the same across all 3 exhibition conditions with slightly more women in groups (Ex. 1: 53.9%, Ex. 2: 54.4%, Control: 58%, see Figure 5a) and slightly more men alone (Ex. 1: 52.3%, Ex. 2: 53.9%, Control: 51.2%). We observed more people in their 20s–50s than in the younger and older age groups; Children were almost always in a group with adults (see Figure 5b).

### 3.2 Observed behaviors

**Figure 6:**
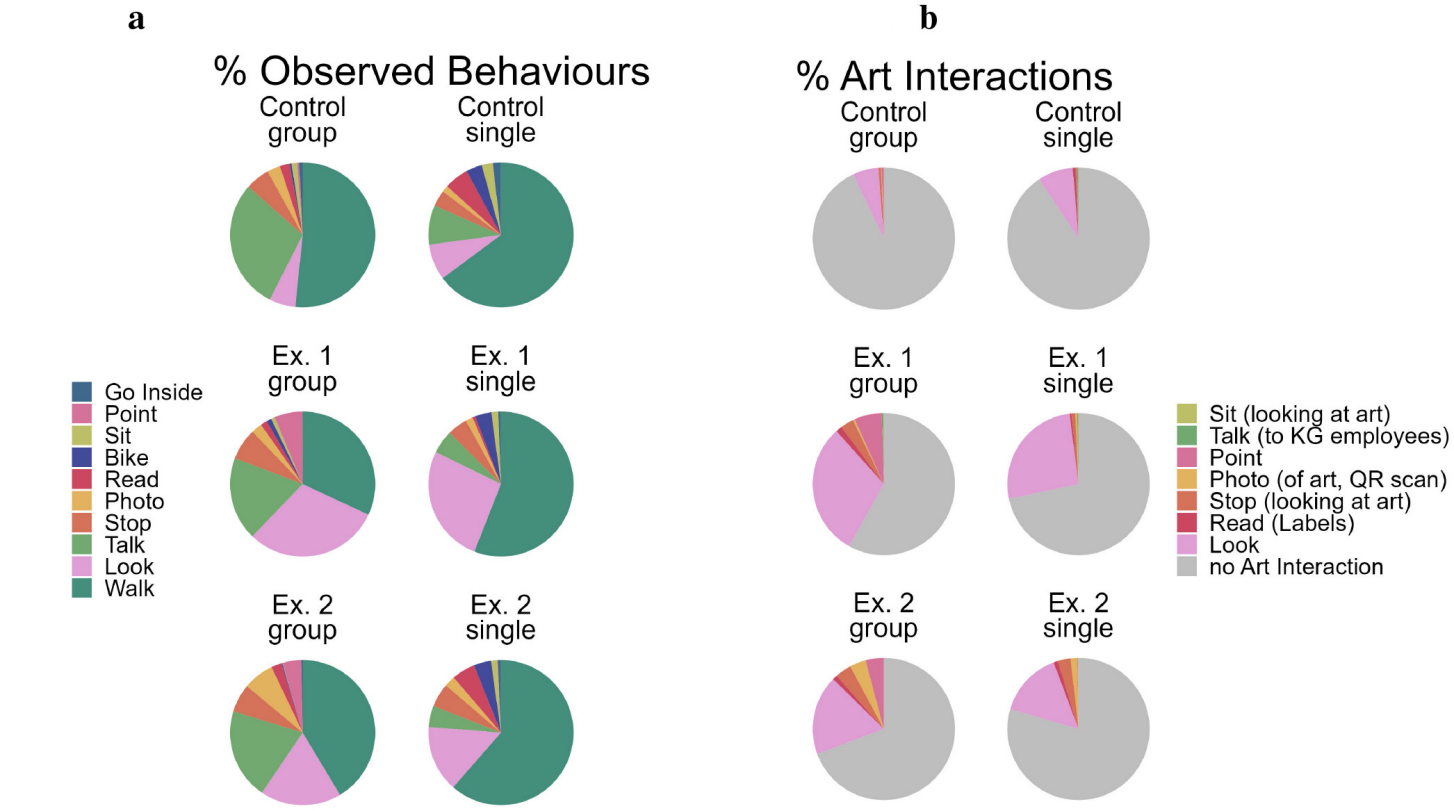
**(a)** All Behaviors; **(b)** only art related.

First, we looked at the visit duration and the mean number of observed behaviors. To then provide an overview of the types and frequency of behaviors that occur in the different exhibition conditions as well as groups and people alone, we looked at the specific behaviors that were observed in more detail. Specifically, we looked at how often each behavior occurred in terms of percentage proportion out of all observed behaviors (see Figure 6a). As some behaviors can occur for art or non-art related reasons, we further split this up into proportions of behaviors done to interact with art specifically vs. all non-art related behaviors (see Figure 6b, as well as Supplementary Figure S5 for occurrence of all modifiers). This however is based on the total number of observed behaviors, not taking into account that some people showed certain behaviors multiple times while others did not show them at all. Thus, to break down the observed behaviors we additionally looked at (1) the percentage of subjects who performed each individual behavior at least once (see Supplementary Figure S3), and (2) how often (if at all) each behavior was observed per subject (see Supplementary Figure S4).

#### 3.2.1 Does the presence of art change how we interact with our urban environment? (exhibition condition)

We observed differences in how people engaged with the study area between our exhibition conditions, indicating that placing art in an everyday environment changes how we interact with it but also that the type of art matters.

On average, the observed passers-by stayed in the study area for slightly less than 1 minute (see Table 2), during which they engaged in 3–4 behaviors on average. However, this differed between exhibition conditions, with the longest time (mean = 0.9 min, SD = 3.99) and highest number of behaviors (mean = 4.01, SD = 3.85) during Ex. 1. Passers-by spent less time (mean = 0.55, SD = 2.05) in the study area during Ex. 2 but engaged in a higher number of behaviors (mean = 3.54, SD = 3.54) than during the control condition (duration: mean = 0.71, SD = 4.23, nr. of behaviors mean = 3.29, SD = 2.88).

**Table 2:** Observation duration (A) and number of observed behaviors (B) per exhibition and single/group condition.

	**Overall**	**Exhibition 1**	**Control**	**Exhibition 2**
**A. Observation duration (in minutes)**				
**Mean (SD)**	0.73 (3.58)	0.9 (3.99)	0.71 (4.23)	0.55 (2.05)
Single person	0.65 (3.37)	0.75 (3.25)	0.66 (4.34)	0.53 (2.25)
Groups	0.92 (4.05)	1.23 (5.32)	0.85 (3.94)	0.59 (1.43)
**[95% CI]**	[0.613, 0.847]	[0.683, 1.109]	[0.432, 0.669]	[0.464, 0.962]
Single person	[0.524, 0.785]	[0.546, 0.961]	[0.381, 0.688]	[0.361, 0.962]
Groups	[0.67, 1.164]	[0.709, 1.748]	[0.435, 0.747]	[0.407, 1.292]
**[Min, Max]**	[0, 109.52]	[0, 85.63]	[0, 109.52]	[0, 32.15]
Single person	[0, 109.52]	[0.02, 40.2]	[0, 109.52]	[0, 32.15]
Groups	[0, 85.63]	[0, 85.63]	[0.03, 62.38]	[0, 14.18]
**B. Number of observed behaviors**				
**Mean (SD)**	3.64 (3.49)	4.01 (3.85)	3.29 (2.88)	3.54 (3.54)
Single person	3.08 (2.75)	3.12 (2.47)	3.03 (2.92)	3.06 (2.89)
Groups	5.04 (4.56)	6.06 (5.4)	3.99 (2.65)	4.75 (4.59)
**[95% CI]**	[3.525 3.753]	[3.801, 4.212]	[3.335, 3.745]	[3.125, 3.464]
Single person	[2.969, 3.182]	[2.967, 3.282]	[2.867, 3.262]	[2.826, 3.231]
Groups	[4.762, 5.317]	[5.535, 6.589]	[4.252, 5.255]	[3.693, 4.288]
**[Min, Max]**	[1, 46]	[1, 42]	[1, 46]	[1, 41]
Single person	[1, 46]	[2, 30]	[1, 46]	[1, 31]
Groups	[1, 42]	[1, 42]	[2, 18]	[1, 41]

0 min likely app crashes where no end was recorded for state behaviors, very long durations = people who work in the area who came back multiple times.

As we see in Figure 4, similar numbers of people pass through the space in all conditions and they typically do so relatively quickly. Adding to this, Figure 6a shows how often each behavior occurred in terms of percentage proportion out of all observed behaviors. In the figure, we see that the most commonly observed behavior across all conditions—*walking*—is not related to art, however tracking *walking* (and *cycling*) establishes a baseline of how many people pass through the space. Here, we can now see that people in the control condition primarily pass through the space, with the main other behavior (for groups at least) being *talking*; this changes when we look at the two art conditions. While *walking* through the space remains the most common behavior (everyone has to arrive and leave in some way), we can observe a change in behaviors in the two art conditions.

Specifically, passers-by not only slow down (at least in Ex.1, see above) but also exhibit additional behaviors other than *walking*. This is not only reflected in the number of tracked behaviors (see above) but also the proportion of behaviors that people engage in, in addition to *walking*, increasing when art is placed in the environment. This change is larger for Ex. 1 than Ex. 2. Differences in interactions with art become even clearer when we look at behaviors that are specifically art related (i.e., *looking, pointing* as well as behaviors that were done for art related reasons e.g., taking a photo of art but not taking a photo of something else; see Figure 6b and Supplementary Figure S5 for occurrence of all modifiers). Overall, more interest was shown toward the KG space during the two exhibitions than during the control (about 90% non art related behaviors, for both groups and people alone), with Ex. 1 (only 58.1% non art related for groups, 71.7% for people who were alone) seeming to be more popular than Ex. 2 (group: 69.3%, single: 79.5% non art related).

Taking a closer look at which behaviors in particular change when art is present, we see that *looking* is the one behavior that stands out. This makes sense, for instance it is unlikely you take a photo of something that you did not look at first and decided that it is something worth taking a photo of.

With regard to behaviors that passers-by can engage in for art or non-art related reasons (see Figure 6b and Supplementary Figure S5), we can for instance observe that during Ex. 2 *photos* seem to not only be more common than in the Control and Ex. 1 (esp. in groups), but people also took more photos of the art than of other things. Similarly, people tended to *stop* more to look at the art than for other reasons in Ex. 2 than people in Ex. 1. However, people who *stop* (or *sit* down) to look at art tend to stop for a shorter time than people who stop or sit down for other reasons (see Supplementary Table S1). However, this may not necessarily reflect how deeply they engage with the art: People may stop more often but for shorter times when looking at art (see Supplementary Figure S4, people stop more than once during exhibitions more often than during the control) and instead walk from one art work to the next. Longer durations while not looking at art may be subjects who sat down to smoke, talk on the phone, eat lunch, wait for someone else, etcetera.

For *reading* we observed similar proportions (for people who were alone) in the Control condition and Ex. 2. This proportion changes when we take the reasons for reading into account: During Ex. 2, a large part of observed *reading* was to read labels, while during the Control condition, it was most likely people reading something on their phone. Note that we cannot make accurate comparisons to Ex. 1 here, as *on the phone* was added as a reason for reading only after Ex. 1.

Statistically, the following three regression models supported the findings above (see Section 2.6): Model 1: Any type of art interaction, Model 2: the most common type of art interaction, i.e., looking, Model 3: any type of art interaction, excluding looking). For all 3 models we found a significant positive effect of exhibition condition on the number of recorded art interactions, meaning that the presences of art matters (see Tables 3, 4). Further, the type of art may matter as this effect seems to be somewhat stronger in Ex. 1 (Model 1: log-mean = 2.37, SE = 0.30, CI = [1.83–3.00], *p* < 0.001; Model 2: log-mean = 2.23, SE = 0.26, CI = [1.76–2.77], *p* < 0.001; Model 3: log-mean = 2.83, SE = 0.68, CI = [1.73–4.50], *p* < 0.001) than Ex. 2 (Model 1: log-mean = 1.76, SE= 0.31, CI = [1.19–2.42], *p* < 0.001; Model 2: log-mean = 1.42, SE = 0.27, CI= [0.91–1.99], *p* < 0.001; Model 3: log-mean = 2.62 SE = 0.68, CI = [1.49–4.29], *p* < 0.001).

**Table 3:** Model 1 results: number of any type of art interaction predicted by exhibition condition and being alone or in a group.

**Predictors**	**Log-mean (SE)**	**CI**	**p**
Intercept	−1.90 (0.29)	−2.52– –1.38	**< 0.001**
Exhibition (Ex. 1)	2.37 (0.30)	1.83–3.00	**< 0.001**
Exhibition (Ex. 2)	1.76 (0.31)	1.19–2.42	**< 0.001**
Condition (single)	−0.00 (0.34)	−0.64–0.70	0.991
Exhibition (Ex. 1): Condition (single)	−1.12 (0.36)	−1.86– –0.45	**0.002**
Exhibition (Ex. 2): Condition (single)	−0.91 (0.37)	−1.68– –0.20	**0.015**
Observations	3,613		
R^2^ Nagelkerke	0.304		

Significant effects are marked in bold type.

**Table 4:** Results for (A) observations of looking (Model 2) and (B) all other art interactions (Model 3) predicted by exhibition condition and being alone or in a group.

**Predictors**	**A. Looking**			**B. All other art interactions**		
	**Log-mean (SE)**	**CI**	**p**	**Log-mean (SE)**	**CI**	**p**
Intercept	−2.09 (0.25)	−2.61– –1.64	**< 0.001**	-3.65 (0.66)	−5.29– –2.58	**< 0.001**
Exhibition (Ex1)	2.23 (0.26)	1.76–2.77	**< 0.001**	2.83 (0.68)	1.73–4.50	**< 0.001**
Exhibition (Ex2)	1.42 (0.27)	0.91–1.99	**< 0.001**	2.62 (0.68)	1.49–4.29	**< 0.001**
Condition (single)	0.03 (0.29)	−0.51–0.63	0.905	−0.21 (0.8)	−1.71–1.60	0.794
Exhibition (Ex1): Condition (single)	−0.91 (0.30)	−1.53– –0.33	**0.003**	−2.27 (0.87)	−4.20– –0.64	**0.009**
Exhibition (Ex. 2): Condition (single)	−0.75 (0.33)	−1.42– –0.12	**0.023**	−1.10 (0.85)	−2.98–0.49	0.194
Observations	3,613			3,613		
R^2^ Nagelkerke	0.242			0.234		

Significant effects are marked in bold type.

Breaking down the observed behaviors further, by looking at (1) the percentage of subjects who performed each individual behavior at least once (see Supplementary Figure S3), and (2) how often (if at all) each behavior was observed per subject (see Supplementary Figure S4), we get a similar picture, with the most likely art interaction being *looking* (see Supplementary Figure S3) and interactions during Ex. 1 being somewhat more likely than during Ex. 2 (with the exception of *taking photos*). Most behaviors were only observed once per person (if at all), however once people interacted with the art they tend to do so more than once (e.g., *looking* and *pointing*, see Supplementary Figures S4C, E).

To sum up, the presence of art changed how people interacted with their environment by increasing the amount of behaviors people are engaged in when they pass through our study space, particularly art interactions such as *looking, pointing, taking photos* of the art etcetera. Overall, we saw more art interaction directed toward Ex. 1 and Ex. 2. As the weather changed from summer to autumn from Ex. 1 to Ex. 2, some differences may be due to this, we therefore analyzed the influence of weather further (see below).

#### 3.2.2 Does being in a group a group vs being alone influence how we interact with our environment (Social Condition)?

In addition to differences between exhibition conditions we observed some slight differences between groups and people who were alone.

First, groups tended to stay in the area longer than people who were alone, across all exhibitions (see Table 2). Overall, groups tended to be more likely to interact with the art than people who were alone (see Figure 6 and Supplementary Figure S3).

Statistically, being alone or in a group does only seem to matter when art is present, as only the interactions with the two art exhibitions were significant in any Models 1–3 (see Tables 3, 4); specifically, when art is present, people in a group seem to be more likely to interact with art than people who are alone. This seems to be primarily groups *looking* at the art (at least for Ex. 2), as the interaction of Ex. 2 and Social Condition is only significant in Model 1 and 2, but not Model 3 (i.e., the Model excluding *looking*). For Ex. 1, *pointing* could be the reason, that all 3 Models show a significant influence of Social Condition; people who were alone own rarely engage in *pointing* behaviors (see Figure 6 and Supplementary Figure S3). Additionally, this and some of the other behaviors may depend on the observed passers-by age, while for other behaviors all ages are similarly likely to show said behaviors. Pointing (see Supplementary Figure S5E), for instance, seems to be more common in groups with children during Ex. 1. For Looking (see Supplementary Figure S5C) on the other hand the age category make up of people for who we observed this behavior is similar to the overall age category make up of all observed people, indicating that people of all ages are similarly likely to look at the art.

#### 3.2.3 Weather influences

We ran three additional regression models, extending the previous models, that include exhibition condition and being alone or in a group as predictors, with temperature as an additional predictor (see Tables 5, 6) of the number or art interactions. These models are mostly in line with our previous models in that both art exhibitions have a significant positive effect on the number of art interactions, however, we additionally see that the influence of being in a group is no longer relevant when we take temperature into account. However, temperature on its own also only has a significant influence on the number of *looking* behaviors (see Table 6A)—this influence is negative. In contrast, if art is present higher temperatures may lead to a higher number of art interactions, at least in Ex. 1. Here we see a significant positive influence of higher temperatures on the number of art interactions (see Tables 5, 6A). Again, this seems to be primarily driven by more *looking* (i.e., the model with all art interactions and the one with only looking) as no significant effects remain in the model that includes only art interactions that are not looking (see Table 6B).

**Table 5:** Model 4 results: number of any type of art interaction predicted by exhibition condition and being alone or in a group, as well as temperature.

**Predictors**	**Log-Mean (SE)**	**CI**	**p**
Intercept	−2.17 (0.36)	−3.00–−1.55	**< 0.001**
Exhibition (Ex1)	2.37 (0.40)	1.66–3.24	**< 0.001**
Exhibition (Ex2)	2.13 (0.61)	0.99–3.39	**< 0.001**
Condition (single)	−0.28 (0.46)	–1.15–0.68	0.537
Temperature	−0.18 (0.10)	–0.39–0.01	0.079
Exhibition (Ex1): Condition (single)	−0.75 (0.52)	–1.81–−0.25	0.148
Exhibition (Ex2): Condition (single)	−0.88 (0.81)	−2.49–−0.68	0.274
Exhibition (Ex1): Temperature	0.23 (0.10)	0.03–0.45	**0.029**
Exhibition (Ex2): Temperature	0.20 (0.14)	−0.06–0.48	0.146
Condition (single): Temperature	–0.09 (0.12)	−0.34–0.16	0.448
Exhibition (Ex1): Condition (single): Temperature	0.08 (0.13)	−0.19–0.33	0.563
Exhibition (Ex2): Condition (single): Temperature	0.05 (0.17)	−0.30–0.39	0.780
Observations	3,613		
R^2^ Nagelkerke	0.327		

Significant effects are marked in bold type.

**Table 6:** Results for (A) observations of looking (Model 5) and (B) all other art interactions (Model 6) predicted by exhibition condition and being alone or in a group, as well as temperature.

**Predictors**	**A. Looking**			**B. All other art interactions**		
	**Log-mean (SE)**	**CI**	**p**	**Log-mean (SE)**	**CI**	**p**
Intercept	−2.45 (0.33)	−3.19–−1.88	**< 0.001**	-3.66 (0.69)	−5.44–−2.57	**< 0.001**
Exhibition (Ex1)	2.37 (0.36)	1.73–3.15	**< 0.001**	2.45 (0.75)	1.19–4.30	**< 0.001**
Exhibition (Ex2)	1.82 (0.59)	0.71–3.02	**0.002**	2.84 (1.02)	0.99–5.08	**0.005**
Condition (single)	-0.18 (0.40)	−0.94–0.67	0.657	-0.60 (522)	−2.60–1.44	0.236
Temperature	−0.22 (0.09)	−0.41–−0.05	**0.015**	-0.02 (0.23)	−0.49–0.50	0.942
Exhibition (Ex1): Condition (single)	-0.65 (0.45)	−1.58–0.21	0.150	-1.58 (1.20)	−4.07–0.82	0.187
Exhibition (Ex2): Condition (single)	−0.87 (0.75)	−2.36–0.59	0.248	−0.63 (1.48)	−3.59–2.31	0.670
Exhibition (Ex1): Temperature	0.26 (0.09)	0.09–0.45	**0.005**	0.09 (0.23)	−0.43–0.57	0.702
Exhibition (Ex2): Temperature	0.22 (0.13)	−0.43–0.57	0.010	0.06 (0.27)	−0.51–0.60	0.836
Condition (single): Temperature	−0.06 (0.11)	−0.27–0.15	0.546	−0.21 (0.28)	−0.82–0.35	0.465
Exhibition (Ex1): Condition (single): Temperature	0.06 (0.11)	−0.17–0.27	0.621	0.15 (0.31)	−0.45–0.80	0.621
Exhibition (Ex2): Condition (single): Temperature	0.00 (0.16)	−0.31–0.31	0.977	0.22 (0.35)	−0.47–0.95	0.533
Observations	3,613			3,613		
R^2^ Nagelkerke	0.267			0.240		

Significant effects are marked in bold type.

Analyzing the influence of temperature separately for each Exhibition condition (see Supplementary Tables S1–S3) shows that temperature only significantly influenced the number or art interactions during the Control. Specifically, higher temperatures made art directed behaviors less likely when no art was present. The influence of temperature was non-significant for both art conditions.

## 4 Discussion

In this observational study, we aimed to investigate how people interact with art in an urban environment in a naturalistic setting. Specifically, we tracked behaviors people were engaged in when passing by a small, publicly visible window gallery in Vienna, comparing two art exhibitions and a no-art control condition. Further, we looked at differences between behaviors in groups and people who were passing through the area alone.

### 4.1 The presence of art changes how we interact with our environment

We observed that people interact with our study area differently when art was present as compared to when no art was present in the KG space. Specifically, we overall observed more interactions with the art space in the art conditions. Passers-by in the no art control did still look at the empty gallery windows occasionally, however, the art clearly captured people's interest and made them more likely to look toward the windows. The main art interaction was simply looking at the KG space, however additional art interactions such as pointing at the art or taking photos of it were also observed. Thus, similarly to Gehl et al. (2006) we conclude that the looks of the city at street level are important. Making the street level more interesting, invites people to interact with the space by slowing down, attracting attention to the art by looking, pointing, or even stopping (although often not for very long, but that appears to be in line with stopping to look at art in museum spaces, e.g., Smith and Smith, 2001; Smith et al., 2017) and taking photos.

Not only that, it may also suggest that while people may generally operate in “life-mode” (Ho and Szubielska, 2025)—a state in which we go about our lives in a pragmatic way to for instance just go from one place to another but in which we may only perceive art around us in a very superficial way—it may be relatively easy to switch to into “art-mode”—a state in which we readily process art found around us in our daily lives. At first glance this may seem to be in contrast to what Ho and Szubielska (2025) suggest; specifically, they see the “art-mode” as a special, relatively rare occurrence in our daily lives. If we, however, consider the location of our study area within Vienna, within walking-distance of multiple museums, in a creative district with residents of likely high cultural capital, it may well be that the passers-by operate in an “art-mode” more readily. Our sample of subjects may have included tourists and residents with higher interest and expertise in art who have already adopted an aesthetic attitude, who are ready to interact with art they encounter in our study area. For the future, it would therefore be necessary to look at art interactions in areas that are overall less conducive to an “art-mode.”

Nevertheless, while people in our specific study area may more readily switch into “art-mode,” for passers-by who only give the artworks a single brief look, we of cannot tell whether they truly switched into an “art-mode” and processed what they saw deeply enough or whether they remained in “life-mode.” However, even a brief glance at an artwork in a window, that may seem rather small, can have lasting impacts. For instance, seeing something we find beautiful in our everyday environments has previously been shown to make us feel calmer and more positive (Knoll et al., 2024)—and beauty can be found in many things in our everyday life, starting from people's pets, to a nice flower, to art placed in a space such as KG. As such, spaces like KG may similarly affect how we feel, and may have the potential to ultimately affect our wellbeing. However, this we cannot see solely based on observations, we get back to this in Section 4.5.

A further aspect missing from our observations is how people interact with the space if other visual material is present in the study space. Our comparisons are between white, empty walls and two different art exhibitions, however people may interact differently from our non-art control and more similarly to our art conditions when other visually interesting material such as posters on upcoming exhibitions, flowers, etc. is present in the space.

### 4.2 Being in a group may make us slightly more likely to interact with art in our environment

While we observed somewhat more art interactions in groups than for people who were alone, these were not particularly strong. Existing differences may simply be due to the nature of being in a group and how this was tracked. We tracked each group as a single subject, meaning for example, if each group member looked at the art only once, this would be a group total of three, whereas a single person would have to actually look three times to get to the same total count of looking. Thus the number of people in a group essentially already increases the chance of any behavior being logged for that group.

That said, the nature of being in a group may make some behaviors genuinely more likely than being alone. This may be non-art related behaviors, e.g., stopping to wait for another group member but also specific art interactions. For example, through pointing behavior (or talking) one member of the group may direct the rest of the group's attention toward the art, while it makes little sense for such behaviors to be observed for someone who is alone.

### 4.3 The type of art placed in our urban environments matters

In contrast, we saw clear differences between the two different art exhibitions. Overall, Exhibition 1 appeared to be more interesting to passers-by than Exhibition 2. Thus, it likely matters not only that art is present but also what kind of art. This may be due to multiple reasons. For one, the artworks in Exhibition 1 are likely easy to see even when walking by at a distance; they have similar sizes and bold colors. The colors may create a contrast to the surrounding, more muted, colors of the buildings at St. Ulrichsplatz, thus making them stand out more. Artworks in Ex. 2, particularly the ones on the left side of the KG space, varied in size, most being relatively small. The colors were somewhat more muted than the colors in Ex. 1 and thus may blend in more with the surroundings. With a larger number of different artworks and settings, this contrast—i.e., in terms of size or color use—may be interesting to systematically study further in future studies.

Another reason for differences between Ex. 1 being more popular may be the content of the art. Ex. 1 featured animals and football, a type of content which, especially given the exhibition time close to the European football championships, may have been easy for people to connect to, or to understand, e.g., by recognizing which football player was depicted. Potentially, Ex. 1 may also simply be a nice, happy thing to see when for instance on the walk home. In contrast, as the art of Ex. 2 explicitly focuses on critiques of capitalism, this may be initially less “happy” and potentially harder to relate to or understand. For example, especially for younger children, they may not “get” the message behind the work. In addition, as many works were a play on popular brands, a casual passers-by may not initially notice that the work reads “crime” instead of “prime” (referring to Amazon Prime). As such, especially due to the smaller size, the art may require people to stop and more closely look at the work to be able to recognize its message. This would be in line with the higher percentage of stopping to look at art, that we observed in this condition. Notably, this kind of engagement may be harder to realize within an everyday life setting where people do not necessarily “seek out” art. Although, some might seek out art in their everyday environment, they are probably less likely to do so than when they choose to go to a museum, for instance. In the case of our study area specifically, we did not observe any behavior that seemed to indicate a targeted visit during our observation—from the way people walked through the area, a majority of the people seemed to pass, then notice, and then engage rather than arriving and directly approaching the observation space. As such, the kind of art in Ex. 2 may require more of a switch to “art-mode” and is not as suited for “smaller” aesthetic experiences that are a part of “life-mode” (see above). More experimental approaches could build on this by more systematically differentiating between different art styles or content.

Taking a broader approach, when considering such art interventions, specifically with regard to their impact not just on passers-by but also on residents. It may be interesting to investigate, based on our results that the kind of art seems to matter, if different art styles, contents, etc. have different impacts specifically for residents or people who are likely to be in the area frequently. Similarly to Kühnapfel et al. (2025) where art was tied specifically to the neighborhood of the exhibition, this may increase connection to said neighborhood and in turn increase interest in the exhibitions. In an ideal case this would be combined with also working together with residents of the neighborhood to also see their needs or wishes for the space—what do they want to see and why?

Further, Estrada Gonzalez et al. (2025)'s findings suggest that street art can have different influences on our perceptions (e.g., safety, friendliness, liking, etc.) of our environment, depending on the type of neighborhood the art is located in. As KG also aims to extend their exhibition space from only their office windows to additional spaces around Vienna, it would be interesting to see whether this extends not only to perceptions of the neighborhood but also to how we interact with the art/neighborhood when the same exhibition is placed in different spaces across the city, with the additional factor of the art being targeted to one neighborhood or the other. This would also allow for assessing to which extent a match between residents needs/wishes for the art and the art exhibition would impact potential outcomes, e.g., in terms of engagement with the exhibition. Which would also allow for an assessment of to what extent findings can be generalized and to what extent they are specific to the combination of exhibition and city/neighborhood context.

### 4.4 Summer time = art time? The potential issue of weather

That said, the difference between Ex. 1 and Ex. 2, may be explained by other factors beyond differences in the art itself. Specifically, weather conditions may have played a role. Observations during Ex. 1 took place at the end of summer with temperatures still being rather high, while Ex. 2 observations took place in autumn when it was chillier and rainier. Research on thermal comfort suggests that how people interact with their environment is influenced by how thermally comfortable they are. Changes in weather will change how much time people spend outdoors, what they do outside, and so on, particularly in autumn (Shooshtarian et al., 2020). Further, thermal comfort may not only be influenced by the weather itself but additional factors such as aesthetics and mood (Knez and Thorsson, 2006). This relation is potentially bidirectional in that how our environment looks may influence how thermally comfortable we are, but also the other way around, the weather may influence how beautiful we find our environment (Eliasson et al., 2007; Knez and Thorsson, 2006; Shooshtarian et al., 2020; Tan et al., 2019).

Temperature appears to have had some sort of influence in our observations, as higher temperatures predicted fewer interactions with our study space when no art was present. With regards to the two art conditions, there may be a small influence of high temperatures in Ex. 1, making art interactions slightly more likely than when no art is present. In summer the KG space at St. Ulrichsplatz often gets a breeze throughout much of the day and offers some shade, thus the space itself may be inviting in summer, but particularly when art was present, people may have felt comfortable to slow down and look at the art during Ex. 1. When people stopped or took photos during Ex. 1 this was often not directed toward the art but for other reasons. Meanwhile in autumn, during Ex. 2 passers-by more often engaged in these behaviors to interact with the art specifically (i.e., stop to look, take photos of it). Thus, people may interact less with the art when the weather is worse, however, the ones who were interested may not have been as easily deterred by the weather (maybe momentarily felt more thermally comfortable) and still stopped or took photos for a closer look at the art. However, for Ex. 2 in particular we can only speculate, as our regression models do not show consistent results, potentially because the temperatures within Ex. 2 were similar throughout, while for Ex. 1 and the Control condition temperatures differed more within observation days.

### 4.5 Limitations and future directions

Unfortunately, we cannot control the weather or the changing of seasons. Within the current study we were unable to perform all observations within the same season, but ideally future studies should compare the same exhibition across seasons or different exhibitions in the same season for somewhat more control over influences of the weather on the behavior of passers-by. A further consideration may also be how place-dependent seasonal differences across different climates, e.g., in a city with an overall hotter climate, autumn may be a more comfortable time to view art in the city while in Vienna it may be Summer. Outside the issue of not being able to control the weather, the observational and exploratory nature of this study comes with a number of additional limitations.

For one, we can only track what we see, we do not know what is happening inside people's minds when they are interacting with the art and can therefore only guess whether they liked it or paid attention to it for other reasons. In a few rare cases we have a pretty good idea whether people liked the art, as we were sometimes able to hear them talk or laugh about it, for example we made a note for a group of three 20-something men to record that one of them exclaimed that the art “is so their vibe” (see Supplementary Figure S7 for a timeline of their visit). However, for most we can only say they showed some sort of interest but not why.

Further limitations were created primarily by human (observers) or technological (observation app) error. First, we tried to track everyone passing by, which worked well with two observers the majority of time. However, when too many people passed through the study area at the same time, we sometimes missed a few people. This issue was further complicated by problems with the tracking app we used. Here, the two main problems were (1) app crashes which result in the need to restart the app and potentially missing people in the meantime; and (2) issues with the data export, where sometimes it took multiple attempts to properly export all data files from the app. Here we want to note that we cannot be 100% sure that all data was exported, there may still be a few missing files or behaviors. Similarly, due to either human error of the observers or failures of the app, there were some mistakes in the data (e.g., a group tracked as a single person), these we typically made notes on during the observations and then manually corrected later on (see osf.io/7gn2r).

These issues could have been at least partly avoided by, for example, filming passers-by and/or picking a different app. However, we did not opt for the first option due to data protection reasons. The app was chosen because of its low financial cost and ease of use; other apps would likely come with different problems. It is probable that some human and technological error would persist even if passers-by were filmed or a different app was used.

Lastly, we did not check for agreement between observers. While we determined before the study how we would track behaviors, we did not formally check whether each observer actually tracked the same subject the same way. We did often talk to each other during observations, for instance, to make sure we coded the right behaviors. In similar future studies, inter-rater reliability should be considered by, for example, having a short period of observation in which all observers track the same rather than different subjects. At the moment the only overlap in tracked participants we may have is due to accidentally tracking the same subjects (again an issue of human error), this however cannot easily be identified in the data and therefore not be used to calculate inter-rater reliability. Indeed, it creates a new limitation in that a few subjects may count double toward our results.

Despite the limitations mentioned above, our findings should be viewed in light of their future potential. Firstly, our results demonstrated that the presentation of art in an urban public space influenced the behavior of passers-by. Displaying artworks slowed people down, encouraged them to stop more, and increased their interaction with the location. This suggests that the presence of art may make public spaces more inviting and engaging. In combination with previous research showing that art can enhance spatial perception (e.g., Estrada Gonzalez et al., 2025), our results suggest that art holds promise for improving both the aesthetics of cityscapes and people's interaction with public spaces. An interesting direction for future research is to examine the types of artworks used. As suggested by our results, different types of artworks may influence behavior differently. Understanding which kinds of artwork, and the experiences they induce, affect specific behaviors could be crucial for urban planning. For example, certain types of art might draw people into the environment more effectively, leading them to pay greater attention to restorative elements, which could in turn contribute to wellbeing, as suggested by previous studies. Conversely, other types of art might be more effective in deterring vandalism. Such nuanced differences are important to consider when thinking about how to implement art in urban public spaces, particularly in terms of the specific outcomes we aim to achieve.

Our study also offers insights for future research aimed at enriching scientific rigor. In particular, we believe that the observational procedure we employed can serve as a valuable tool to complement other methodologies in highly ecological settings. For example, in previous urban intervention studies, participants are often aware that they were being observed (e.g., Kühnapfel et al., 2025; Mikuni et al., 2024; Szubielska et al., 2024), sometimes even through devices such as mobile eye trackers (e.g., Dehove et al., 2024; Mitschke et al., 2017; Chana et al., 2023). While such methods are valuable for examining attention patterns and the impact of interventions, the awareness of being tracked may itself alter participants' natural behavior. In contrast, the observational method introduced in the present study provides an opportunity to capture more authentic behavior and interaction. It can help answer questions such as: Do observed behaviors align with those seen in more controlled, less naturalistic settings? Do people actually interact with the specific environmental features targeted by urban aesthetic interventions? Furthermore, observational data can inform more targeted research by identifying which environmental elements people naturally engage with. This can help urban designers and researchers determine which features might be leveraged or improved in aesthetic interventions aimed at promoting well-being or other outcomes.

One of the main advantages of observational methods is their ease of implementation. They are relatively simple to set up and can be adapted to a wide range of environments with minimal equipment and only minor adjustments once an observation protocol is established. This flexibility is particularly valuable in dynamic urban settings, where naturally occurring changes can be studied more quickly and opportunistically than in controlled intervention studies, which often require extensive planning and coordination (e.g., with city authorities). Ultimately, combining observational methods with other research approaches can support the design of urban environments that promote human thriving. Observations can reveal how people interact with their surroundings in everyday life, while experimental and physiological studies can provide insight into how those interactions impact wellbeing. Together, we hope our study will inspire further research on the role of art in public spaces, highlighting its potential to address pressing societal challenges, including the enhancement of urban public spaces.

## 5 Conclusion

Our observational study shows that people interact with art that is publicly accessible from street level in an everyday urban environment naturally. Most commonly by looking at it when passing through the area in which art is presented. However, people may also slow down or even stop to look at the art and interact with it further. While we do see that people clearly interact with the art, the exploratory and observational nature of this study limits our conclusions both in terms of not knowing how people actually feel about the art (we cannot observe what is happening inside people's minds) but also in terms of the observational procedure itself. We thus, see the need to improve the observational method: We may extend our observations into additional spaces; include an additional condition that present visual material other than art (e.g., exhibition posters, flowers, etc.) or additional art that may be more tied to the space it is placed in content-wise; combine observational and non-observational data to benefit from seeing both how interactions occur naturally but to also be able to see how people feel about and are influenced by such interactions.

Ultimately we hope showing that people do interact with such spaces naturally, combined with studies that show such spaces have positive effects on our our wellbeing, the look of the city, community and so on, will help to further increase support and implementation of such spaces.

## Data Availability

The datasets presented in this study can be found in online repositories. The names of the repository/repositories and accession number(s) can be found below: https://osf.io/wh5uz/.
