# Supplementary material for: Looking at people looking at art: observations of art interactions in an everyday urban environment
Source: Front Psychol. 2025 Sep 8;16:1658946. doi: 10.3389/fpsyg.2025.1658946 (PMC12450967; doi:10.3389/fpsyg.2025.1658946)
Supplement: Supplementary file 1 [file Data_Sheet_1.pdf]

frontiers

Supplementary Material

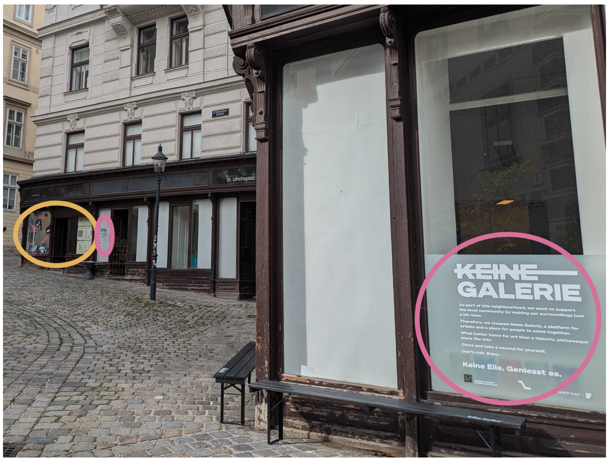

Figure S1. Art at neighbouring office space and labels at Keine Galerie. During the Control Exhibition labels (pink) explaining what KG is and artwork in the neighbouring office space (yellow) remained.

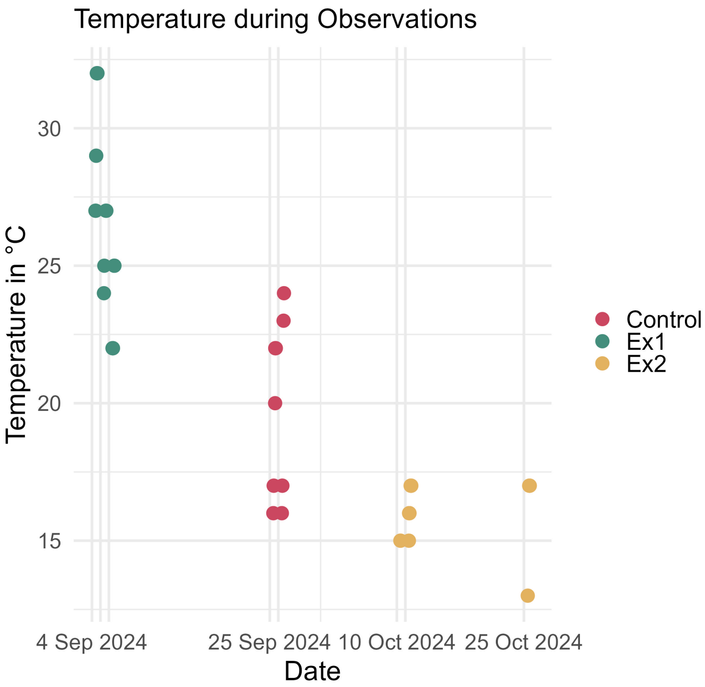

Figure S2. Temperature during observations. We recorded temperatures every time we started a new observation session in Behayve. Mutltiple sessions were started throughout the day due to lunch breaks and app crashes. Temperatures decreased from Ex1 to the Control condition and again between Control and Ex2. Additionally, during the first day of Ex2 it started raining which led us to stop observations early that day.

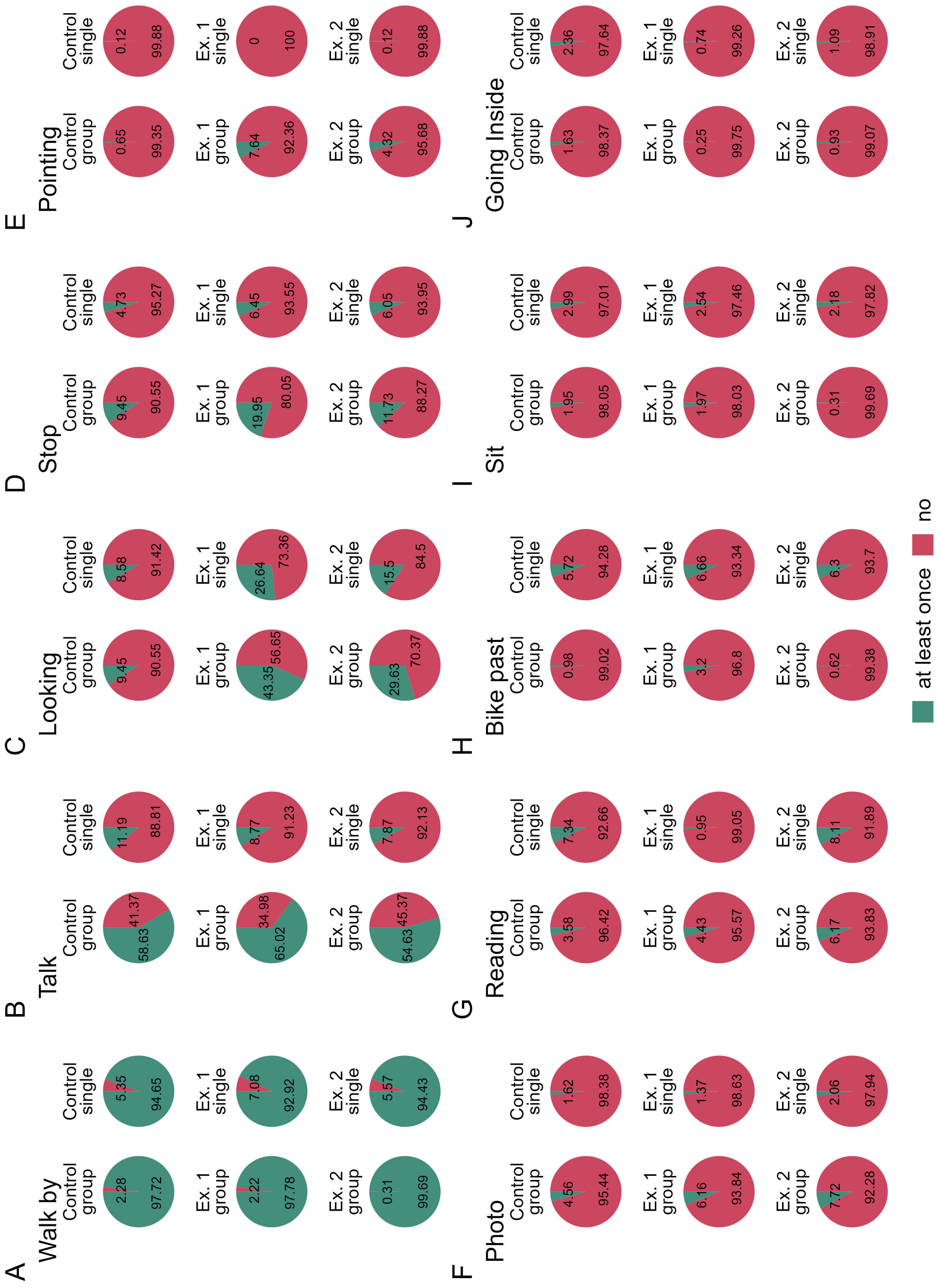

Figure S3. Percentage of observed people (out of all people in during that exhibition and single/group condition) who did each behaviour at least once (green) vs percentage of people who did not do the behaviours at all (red).

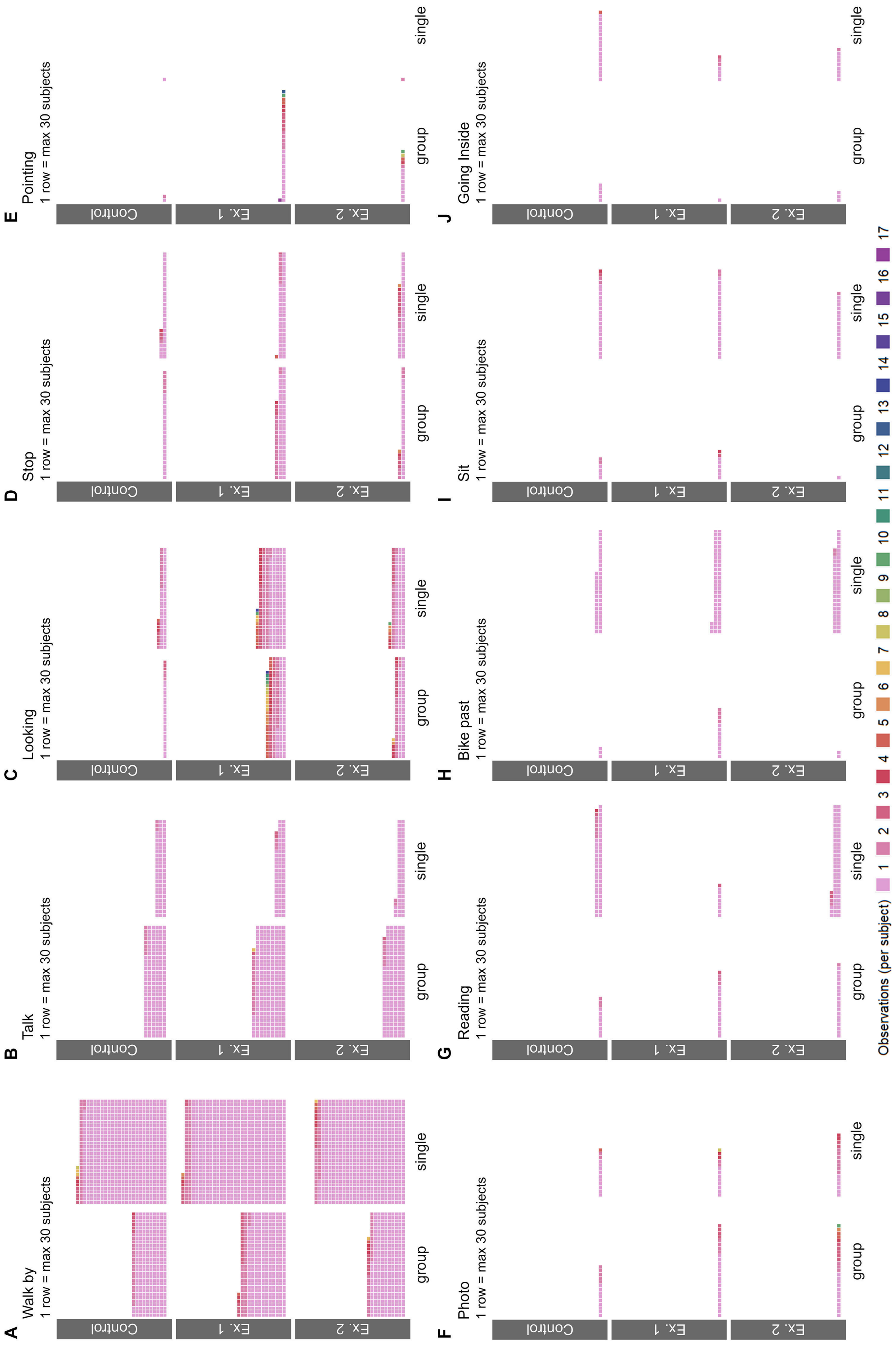

Figure S4. Number of observations of each behaviour for each subject who did the corresponding behaviour once or more.

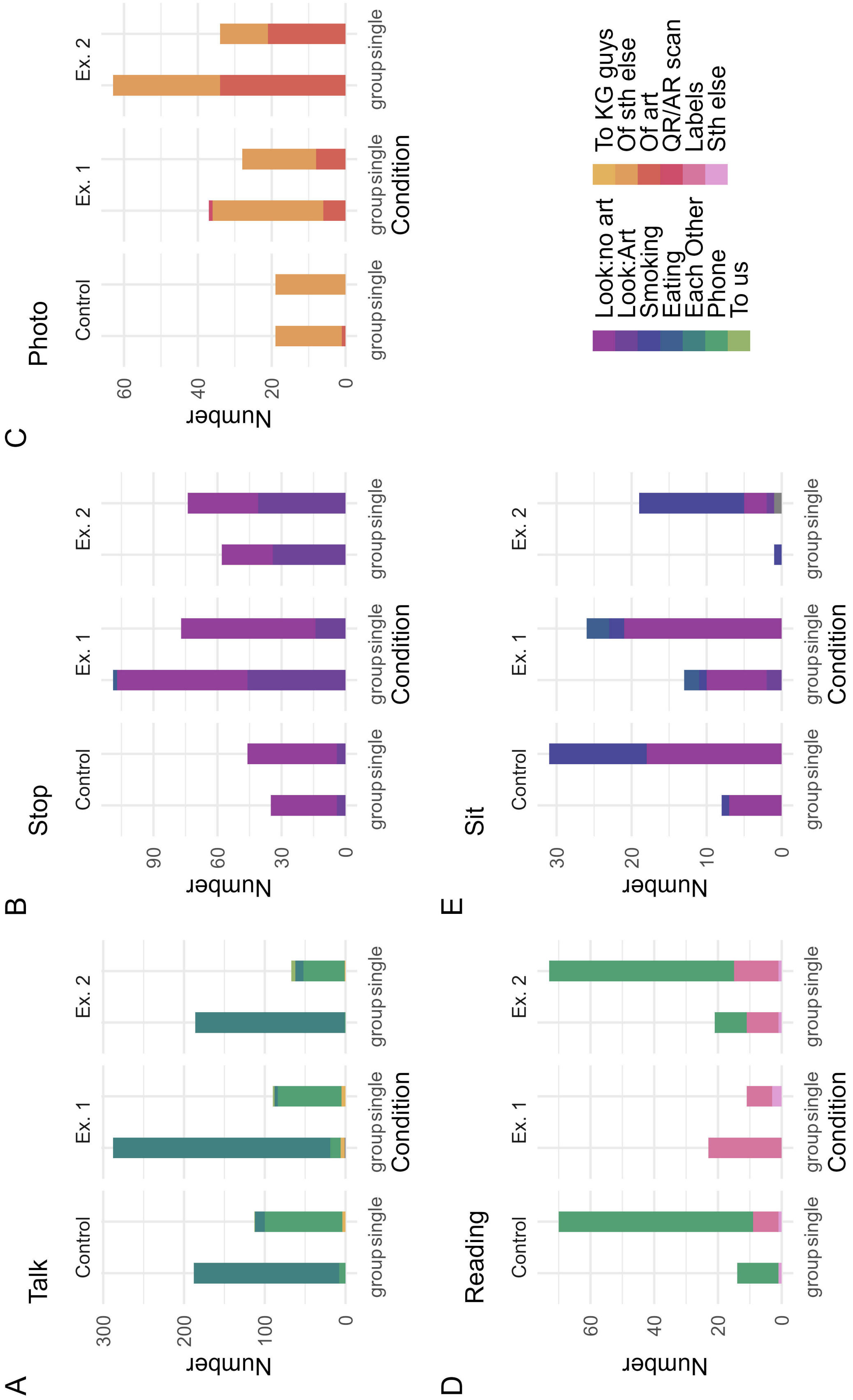

Figure S5. Modifiers to Behaviours and how often they were observed

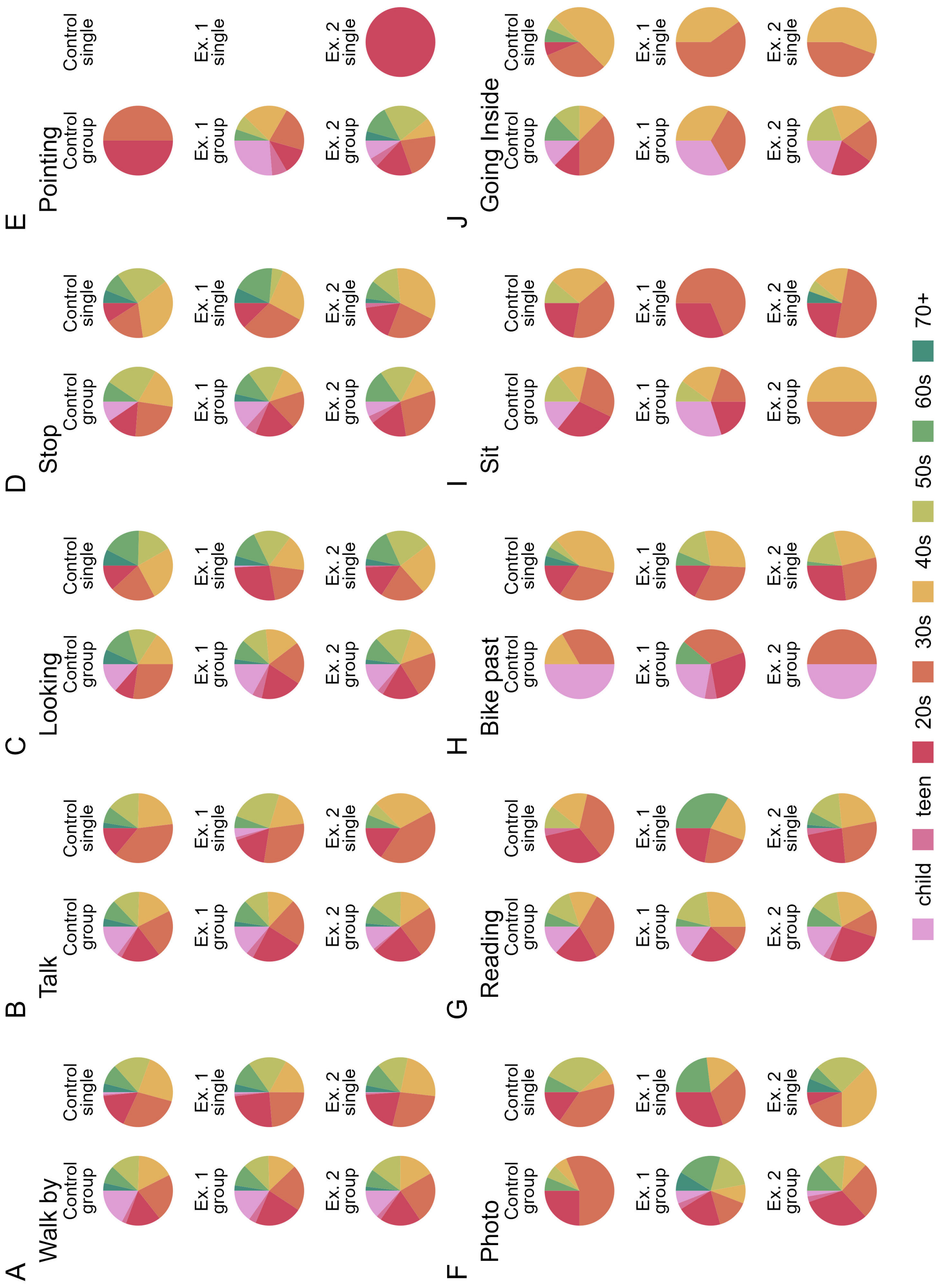

Figure S6. Age distributions for each behaviour to see whether any behaviour is more likely to be performed by any specific age group.

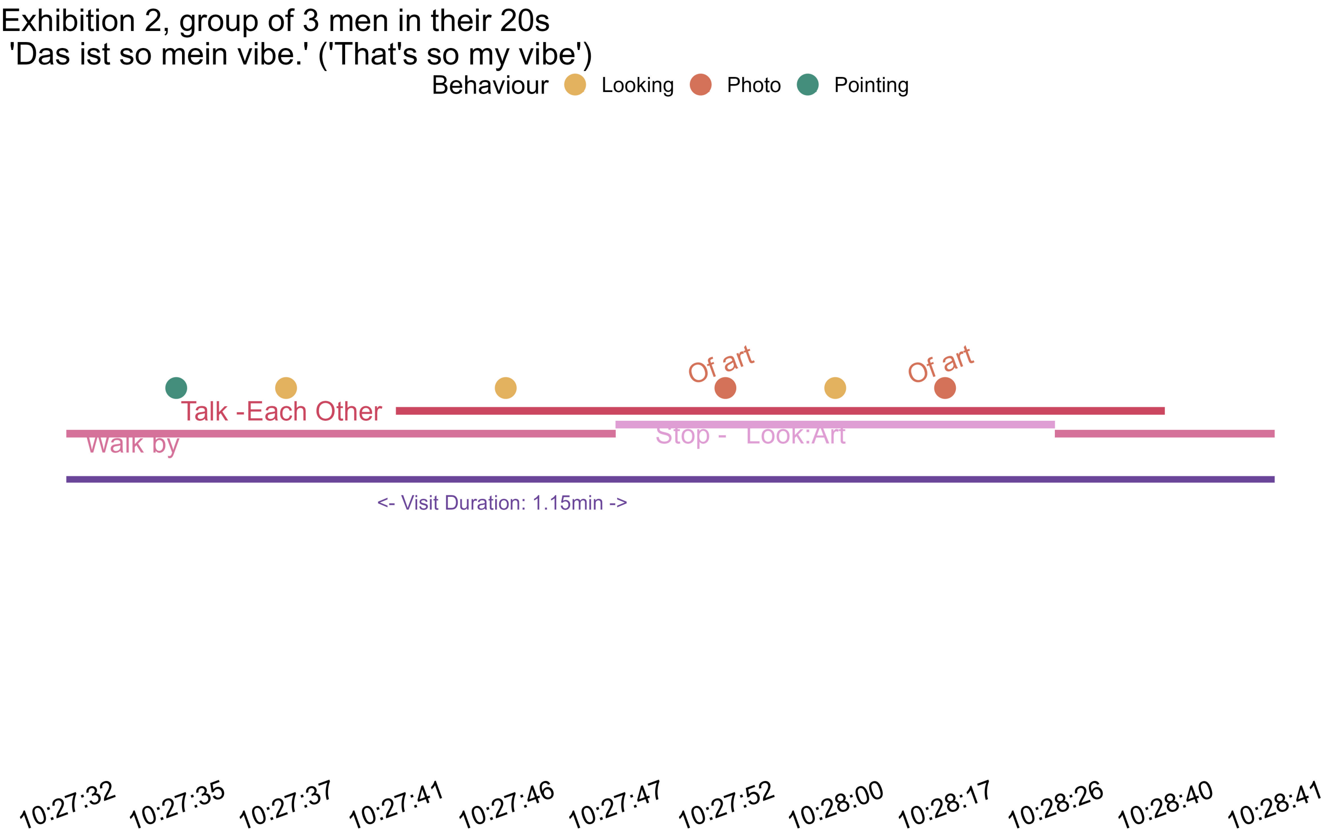

Figure S7. Example of an observation timeline for a group of 3 men passing through the study space during Ex. 2. One group member exclaimed that the art ’is so his vibe’.

Table S1. Durations of people stopping or sitting down to look or not look at art. It appears that people tend to stop or sit down for a longer time when not looking at art than when looking at art. This may not necessarily reflect how deeply they engage with the art: People may stop more often but for shorter times when looking at art and instead walk from one art work to the next. Longer durations while not looking at art may be subjects who sat down to smoke, talk on the phone, wait for someone else etcetera.

**A) Duration stopping or sitting down (in seconds)**		
	**looking at art (n = 146)**	**not looking at art (n = 350)**
Mean (SD)<br>[95% CI]	31.72 (56.31)<br>[16.04, 47.39]	116.82 (259.44)<br>[74.53, 159.11]
**B) Duration stopping only (in seconds)**		
	**looking at art (n = 143)**	**not looking at art (n = 256)**
Mean (SD)<br>[95% CI]	30 (56.77)<br>[13.87, 46.14]	45.65 (154.01)<br>[15.25, 76.05]
**C) Duration sitting only (in seconds)**		
	**looking at art (n=3)**	**not looking at art (n = 94)**
Mean (SD)<br>[95% CI]	74.55 (1.77)<br>[58.67, 90.43]	273.09 (359.65)<br>[166.28, 379.89]

Table S2. GLM Results for all art interactions with separate models per exhibition condition (A. Ex1, B. Ex2, C. Control), including temperature as predictor

		Intercept	Temp.	Cond. (Single)	Temp.: Cond. (Single)	Obs.	R^2 Nagelkerke
**A. Ex. 1**	*Log-Mean (SE)*	0.46 (0.08)	0.05 (0.03)	-1.13 (0.12)	-0.02 (0.04)	1352	0.261
	CI	0.30 – 0.62	-0.00 – 0.10	-1.37 – -0.89	-0.10 – 0.06		
	p	<**0.001**	0.052	<**0.001**	0.644		
**B. Ex. 2**	*Log-Mean (SE)*	-0.14 (0.13)	0.02 (0.11)	-0.92 (0.19)	-0.05 (0.14)	1150	0.111
	CI	-0.42 – 0.11	-0.18 – 0.25	-1.29 – -0.54	-0.33 – 0.23		
	p	0.292	0.853	<**0.001**	0.748		
**C. Control**	*Log-Mean (SE)*	-2.02 (0.22)	-0.18 (0.07)	-0.20 (0.27)	-0.09 (0.08)	1111	0.129
	CI	-2.48 – -1.63	-0.32 – -0.05	-0.72 – 0.34	-0.26 – 0.08		
	p	<**0.001**	**0.010**	0.453	0.267		

Table S3. GLM Results for looking with separate models per exhibition condition (A. Ex1, B. Ex2, C. Control), including temperature as predictor

		Intercept	Temp.	Cond. (Single)	Temp.: Cond. (Single)	Obs.	R^2 Nagelkerke
**A. Ex. 1**	*Log-Mean (SE)*	0.14 (0.08)	0.04 (0.02)	-0.88 (0.11)	-0.01 (0.04)	1352	0.148
	CI	-0.01 – 0.29	-0.01 – 0.09	-1.09 – -0.67	-0.08 – 0.06		
	p	0.067	0.094	<**0.001**	0.798		
**B. Ex. 2**	*Log-Mean (SE)*	-0.67 (0.12)	0.01 (0.09)	-0.72 (0.16)	-0.06 (0.12)	1150	0.057
	CI	-0.91 – -0.45	-0.16 – 0.20	-1.03 – -0.41	-0.29 – 0.16		
	p	<**0.001**	0.940	<**0.001**	0.606		
**C. Control**	*Log-Mean (SE)*	-2.27 (0.23)	-0.22 (0.07)	-0.12 (0.28)	-0.06 (0.09)	1111	0.135
	CI	-2.76 – -1.86	-0.37 – -0.08	-0.65 – 0.45	-0.23 – 0.11		
	p	<**0.001**	**0.003**	0.657	0.456		

Table S4. GLM Results for all art interactions except looking with separate models per exhibition condition (A. Ex1, B. Ex2, C. Control), including temperature as predictor

		Intercept	Temp.	Cond. (Single)	Temp.: Cond. (Single)	Obs.	R^2 Nagelkerke
**A. Ex. 1**	*Log-Mean (SE)*	-0.83 (0.14)	0.07 (0.05)	-2.47 (0.35)	-0.05 (0.12)	1352	0.287
	CI	-1.12 – -0.56	-0.02 – 0.16	-3.22 – -1.83	-0.30 – 0.17		
	p	<**0.001**	0.112	<**0.001**	0.648		
**B. Ex. 2**	*Log-Mean (SE)*	-1.03 (0.22)	0.04 (0.17)	-1.30 (0.33)	0.01 (0.26)	1150	0.102
	CI	-1.49 – -0.64	-0.27 – 0.42	-1.97 – -0.65	-0.50 – 0.54		
	p	<**0.001**	0.823	<**0.001**	0.961		
**C. Control**	*Log-Mean (SE)*	-3.65 (0.42)	-0.02 (0.15)	-0.43 (0.55)	-0.21 (0.18)	1111	0.036
	CI	-4.61 – -2.92	-0.31 – 0.29	-1.52 – 0.71	-0.59 – 0.15		
	p	<**0.001**	0.910	0.440	0.259		
